# Polymer based dual drug delivery system for targeted treatment of fluoroquinolone resistant *Staphylococcus aureus* mediated infections

**DOI:** 10.1038/s41598-023-38473-3

**Published:** 2023-07-14

**Authors:** Gopalakrishnan Thamilselvan, Helma David, Anusree Sajeevan, Shobana Rajaramon, Adline Princy Solomon, Ramya Devi Durai, Vedha Hari B. Narayanan

**Affiliations:** 1 Quorum Sensing Laboratory, Centre for Research in Infectious Diseases (CRID), School of Chemical and Biotechnology, SASTRA Deemed to be University, Thanjavur, 613401 India; 2 Pharmaceutical Technology Laboratory, School of Chemical and Biotechnology, SASTRA Deemed to be University, Thanjavur, 613401 India

**Keywords:** Microbiology, Health care

## Abstract

The present study attempts to treat *S. aureus-induced* soft skin infections using a combinatorial therapy with an antibiotic, Ciprofloxacin (CIP), and an efflux pump inhibitor 5-Nitro-2-(3-phenylpropoxy) pyridine (5-NPPP) through a smart hydrogel delivery system. The study aims to reduce the increasing rates of infections and antimicrobial resistance; therefore, an efflux pump inhibitor molecule is synthesized and delivered along with an antibiotic to re-sensitize the pathogen towards antibiotics and treat the infections. CIP-loaded polyvinyl alcohol (PVA) hydrogels at varying concentrations were fabricated and optimized by a chemical cross-linking process, which exhibited sustained drug release for 5 days. The compound 5-NPPP loaded hydrogels provided linear drug release for 2 days, necessitating the need for the development of polymeric nanoparticles to alter the release drug pattern. 5-NPPP loaded Eudragit RSPO nanoparticles were prepared by modified nanoprecipitation—solvent evaporation method, which showed optimum average particle size of 230–280 nm with > 90% drug entrapment efficiency. The 5-NPPP polymeric nanoparticles loaded PVA hydrogels were fabricated to provide a predetermined sustained release of the compound to provide a synergistic effect. The selected 7% PVA hydrogels loaded with the dual drugs were evaluated using Balb/c mice models induced with *S. aureus* soft skin infections. The results of in vivo studies were evidence that the dual drugs loaded hydrogels were non-toxic and reduced the bacterial load causing re-sensitization towards antibiotics, which could initiate re-epithelization. The research concluded that the PVA hydrogels loaded with CIP and 5-NPPP nanoparticles could be an ideal and promising drug delivery system for treating *S. aureus*-induced skin infections.

## Introduction

*Staphylococcus aureus* is a highly adaptable and tenacious pathogen known to cause a wide range of infections. The survival characteristics of the pathogen in different host niches are due to their ability to produce various pathogenic determinants. Although there lies the first line of antibiotic treatment regimens, the pathogen defends at various levels to cause the waves of resistance—a silent pandemic “AMR”. Notable strategies are through the protective layer, the biofilm that enables the inter-/intra species communication (Quorum Sensing; QS) and emerges as a battleground to withstand host-immune response and antibiotic pressure. The current study attempts to resensitize the fluoroquinolone-resistant strain to the same class of antibiotics on inhibiting NorA efflux pump using a suitable drug delivery system.

Hydrogels are three-dimensional swellable structures synthesised by chemical or physical crosslinking methods. Due to their inherent nature and unique properties, such as detainment of water, glassy structure, and sensitivity towards environmental triggers, they are widely used in the biomedical field to treat various diseases through site-specific applications. Hydrogels are explored as favourable substrates applied in tissue engineering for transplantation of cells and wound healing, biosensors and coatings, and drug delivery vehicles^1–3^. Hydrogels functionalized with specific properties are found to replace or treat tissues and organs by interacting with biological systems.

Polyvinyl alcohol (PVA) is a synthetically prepared water-soluble polymer, capable of forming an insoluble hydrogel matrix when cross-linked using chemical or physical methods^4–6^. The advantage of using PVA is based on its nature of biocompatibility, non-toxicity, transparency, and biodegradability^7–9^. In today’s scenario, PVA hydrogels are being explored with or without adding other polymer composites and polymeric nanoparticles by loading different categories of drugs and validating them for the treatment of various infections^10–12^. PVA-based hydrogels are recognized widely as they could be fabricated with the required stability, swelling index, and drug release pattern^13–15^.

Ciprofloxacin hydrochloride (CIP), belonging to the family of Fluoroquinolone class, is a broad-spectrum chemically synthesized antibiotic known to treat a wide range of infections caused by gram-positive and gram-negative pathogens^9^. It targets bacterial DNA gyrase and is commonly used for treating skin infections and ocular infections^16–18^. One of the limitations of the drug is the low half-life (approximately 4 h); therefore, sustained or prolonged effects of the drug could not be achieved by conventional therapy^19–21^. A recent study involving the loading of CIP into PLGA microspheres in combination with inhibitor ginsenoside Rh2 for treating *S. aureus* infections showed sustained release of the drug and was validated through in vivo studies^22^. The studies on the combination of cyclodextrin with agar in PVA hydrogels to deliver 3-methyl benzoic acid (3-MBA) to treat infections occurring on the skin and mucosa were found to demonstrate synergistic and sustained release profile of CIP and 3-MBA for > 1 week^23^. Another study on keratose hydrogels loaded with CIP provided promising results in treating and inhibiting *Pseudomonas aeruginosa* infections on wounds for 10 days with a reduction in bacterial load and enhanced wound healing properties^24^. The reported studies have proved that adding adjuvant molecules with CIP showed synergistic effects with minimum antimicrobial resistance for treating infections.

The 5-nitro-2-(3-phenylpropoxy) pyridine (5-NPPP) is a chemically synthesized compound through a bio-isosteric approach. It was identified and proved to be a promising efflux pump inhibitor and an inhibitor of biofilm formation, as observed through the results of in vitro studies. The synergistic efficacy of the compound was explored in combination with Ciprofloxacin for treating *S. aureus*-mediated Soft Skin Infections (SSIs)^25^. Nanotechnology advancements have helped researchers explore it as a medium for drug delivery applications. The unique features of polymeric nanoparticles as drug delivery carriers are non-toxicity, modified or prolonged drug release, site-specific delivery, solubilization and stabilization of poorly soluble drugs, and improvisation of bioavailability^26–28^. Nanoparticles with a limited dose of drugs can also be directed to solve problems such as antibiotic resistance, multidrug efflux pumps, antibiotic degradation, and biofilm formation inhibition^29,30^. Earlier studies have proved that using nanoparticles as drug delivery carriers could act upon the target effectively, thereby reducing the rates of antimicrobial resistance, increasing higher chances of re-epithelization in wounds, and delivering antibacterial agents effectively^31^. Further taking a step forward, researchers have developed the hydrogel matrix dispersed with drug-loaded nanoparticles, which have been reported to be effective in treating bacterial infections topically^32^.

Based on the above background study, the current work involves the design of topical hydrogels loaded with dual molecules, namely CIP (free drug) and 5-NPPP (as polymeric nanoparticles), for treating SSIs in a combinatorial approach. The key criterion of the work is based on the characteristic nature of the drugs, the selection of polymers, and their concentrations. Further, the hydrogels were formulated with the optimal composition of the polymer that could provide a sustained release profile for topical delivery of both drugs for a period of 5 days and was selected based on the in vitro studies. The selected smart delivery system was evaluated by in vivo studies to provide the maximum therapeutic potential and promising treatment to tackle bacterial soft skin infections caused by *S. aureus.*

## Results and discussion

### Drug loading in the hydrogels

The hydrogels loaded with CIP and 5-NPPP were individually evaluated for drug present by crushing each hydrogel and releasing the loaded drug into buffer solutions overnight. The filtered solutions were analyzed by UV–Visible spectrophotometer and were estimated for the concentration of the drug using the standard calibration data showed the individual drugs at 98 ± 2% in each hydrogel, depicting 0.96–1.0 mg CIP and 5-NPPP per hydrogel, individually (Table 1).

**Table 1:** Drug loading in hydrogels.

PVA concentration (% w/v)	Drug loading (%)	
	Ciprofloxacin loaded hydrogels	5-NPPP nanoparticles loaded hydrogels
5	100.7 ± 0.7	99.4 ± 0.8
7	101.3 ± 1.2	98.8 ± 0.3
9	100.8 ± 0.5	100.1 ± 1.9
11	100.6 ± 0.8	99.9 ± 1.6

### In vitro drug release profile of CIP-loaded hydrogels

**Figure 1:**
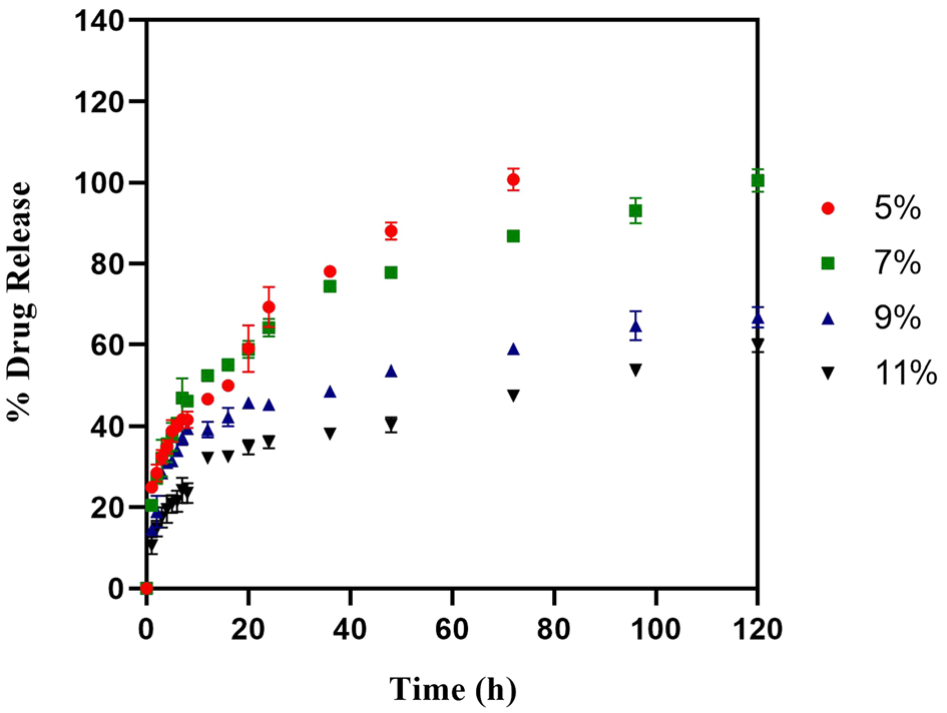
In vitro drug release of Ciprofloxacin from Ciprofloxacin loaded hydrogel. This shows the cumulative amount of ciprofloxacin hydrochloride being released over a period of 120 h (n = 3, p < 0.05).

The in vitro release pattern of the hydrogels was studied for 5 days, with the conceptual idea of selecting and optimizing the hydrogels to provide a linear and complete release of the loaded drugs in the given therapeutic application period for soft skin infections wound healing. In phosphate buffer pH 7.4, the in vitro release profile of CIP-loaded hydrogels prepared with PVA at 5, 7, 9, and 11% w/v displayed cumulative maximum drug release of 100% (72 h), 100% (120 h), 66% (120 h) and 59% (120 h), respectively. The hydrogels with a low concentration of polymer (5% w/v PVA) exhibited a higher swelling index and faster disintegration. They showed maximum drug release within 3 days, whereas the hydrogels containing 7–11% w/v showed reduced swelling and a more sustained release profile for 5 days. However, in the case of 9% and 11% PVA hydrogels loaded with CIP, the release profile demonstrated poor linearity and could not reach maximum drug release at the end of 5 days as shown in (Fig. 1). Correspondingly, a study by Sharma et al*.* demonstrated the loading of CIP onto bi-functionalized PEG glyoxylic aldehyde-chitosan cross-linked hydrogels at various concentrations such as 3%, 6%, 9%, and 12% w/v of polymer, wherein hydrogels with high polymer concentration displayed more sustained release of CIP (80% at 24 h) compared to lower polymer concentration (95% at the end of 10 h)^33^. In another study, the CIP-loaded hydrogels polysaccharide polymers such as carboxy methyl guar gum and gelatin aided the faster release (85%) of CIP within 16 h^34^. Our results were in agreement with the findings of Yang et al*.*, which showed the drug release of CIP from electrospun Chitosan/PVA/Graphene oxide membrane where the linearity was not matched due to the distribution of the drug in a strong network structure and low swelling of nanofibers demonstrating the low release of CIP after 168 h^35^. Henceforth, from our experiments, the 7% PVA hydrogels of CIP were considered ideal to provide the intended linear sustained release profile for 120 h as shown in (Fig. 1). A similar pattern of drug release was demonstrated by Zhou et al*.*, where the research findings of porous PVA scaffold loaded with ciprofloxacin with varying %w/v concentrations of 10%, 14%, and 18% were found to show cumulative drug release of 86.0%, 76.6%, and 54.5% respectively at the end of 5 days (128 h)^36^, proving that the reduced drug release was observed with increase in the concentration of PVA.

As the current study focuses on dermal application to treat skin wound infections, the in vitro release experiments were carried out using phosphate buffer pH 5.8 to mimic the normal skin pH (4.0–5.9) condition^37,38^. In the case of phosphate buffer pH 5.8 media, although an analogous pattern of sustained release was maintained for 5 days, the initial burst release of CIP was higher (40–50%) within 1–2 h, followed by linear slow release of the remaining drug (Supplementary Fig. 1). This could be attributed to the pH-dependent increase in the solubility of CIP in an acidic environment^39^. Overall, in the present study, a promising trend of CIP sustained release was demonstrated for its prolonged beneficial efficacy, wherein the hydrogels loaded with a minimum dose of CIP (1 mg/hydrogel) have been proven to be effective in providing a sustained release profile to reach the maximum of ~ 95–99% drug for 5 days. This phenomenon could be attributed to increased stability and crosslinking by the high molecular weight of the polymer PVA^40,41^.

### In vitro drug release profile of 5-NPPP loaded hydrogels

**Figure 2:**
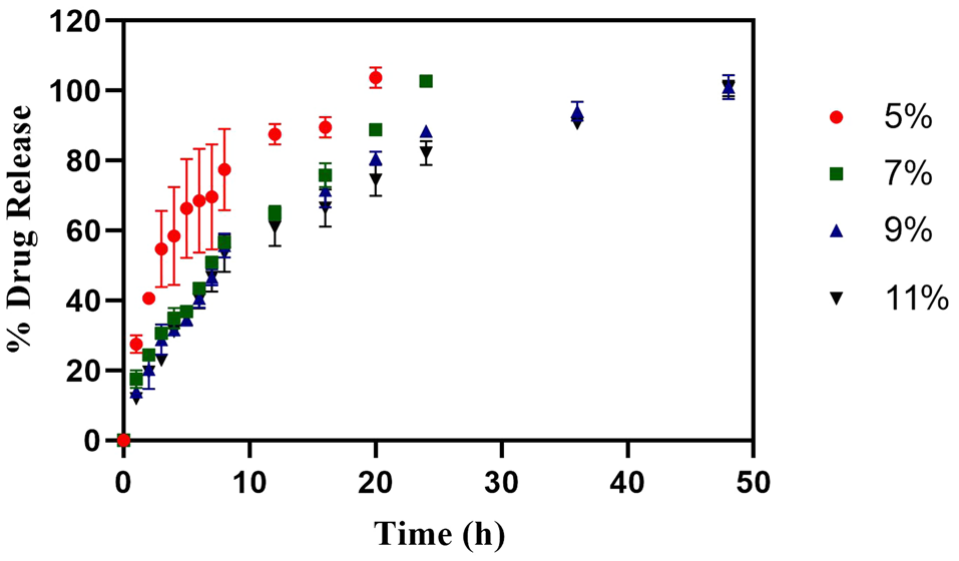
In vitro drug release of 5-NPPP loaded hydrogel. This shows the cumulative amount of 5-NPPP being released over a period of 50 h (n = 3, p < 0.05).

On the other hand, the in vitro release profile of pure 5-NPPP loaded hydrogels exhibited sustained release of the drug in the phosphate buffer pH 7.4 for the maximum period of 2 days. The hydrogels prepared with a low concentration of PVA as 5% and 7% w/v displayed cumulative maximum drug release at 20 h and 24 h, respectively. When the polymer concentration was increased (9% and 11% w/v), the drug release was sustained to reach 100% at 48 h due to a reduction in the swelling index as shown in (Fig. 2). However, the release profile could not be complemented with the CIP-loaded hydrogels to provide a pre-determined release pattern for the expected synergistic effects. To improve the stability of 5-NPPP and optimize the required sustained release pattern for 5 days, the development of polymeric nanoparticles for 5-NPPP, followed by its loading in the hydrogels was intended.

### Development of polymeric nanoparticles of 5-NPPP and entrapment efficiency studies

An analogous trend sustained the release of both CIP and 5-NPPP for 5 days in the desire to provide a synergistic and combinatorial effect of the two molecules to reduce antibacterial resistance and improve the therapy. Since the 5-NPPP-loaded hydrogels provided a sustained release for a period of 2 days only, the development of a particulate drug delivery system was intended, which could be loaded into the hydrogels to provide a dual sustained release effect. The entrapment efficiency of the 5-NPPP—Eudragit RSPO polymeric nanoparticles prepared with different ratios of the drug:polymer as 1:0.5, 1:1, and 1:2 was found to be 65.20 ± 2.55; 90.03 ± 0.50 and 85.5 ± 4.80, respectively (Table 2). An increase in the polymer concentration from 1:0.5 to 1:1 had shown a notable increase in the entrapment of the molecules, as an equal/higher amount of polymer molecules was necessary to encapsulate the drug molecules to form stable particles. However, a further increase in the polymer concentration (1:2 ratio) displayed no significant difference in the entrapment of the drug. The results were comparable to the previously published reports of Eudragit polymeric nanoparticles, wherein an equal ratio of drug and polymer showed maximum entrapment efficiency^42,43^. Hence, the nanoparticles developed with a 1:1 ratio with maximum drug entrapment of 90% were selected as the optimized ones for further characterization and application.

**Table 2:** Physico-chemical characterization of 5-NPPP-Eudragit RSPO polymeric nanoparticles.

5-NPPP:Eudragit RSPO ratio	Size (nm)	PDI	Zeta Potential (mV)	Entrapment Efficiency (%)
1:0.5	269	0.279	+ 6.47	65.20 ± 2.55
1:1	281.7	0.239	+ 8.39	90.03 ± 0.50
1:2	234.8	0.229	+ 6.99	85.5 ± 4.80
1:1 (after 6 months storage at 30 °C/65%RH	295	0.230	+ 7.57	89.5 ± 0.50

### Zeta sizer analysis of 5-NPPP polymeric nanoparticles

The average particle size, particle distribution (poly dispersity index PDI), and the zeta potential of the formulated nanoparticles are shown in Table 2. The nanoparticles prepared with different ratios of 5-NPPP:Eudragit RSPO (1:0.5, 1:1, and 1:2) exhibited average particle size in the narrow range of 230–280 nm with the PDI value of 0.229–0.279, indicating uniform monodisperse nature of the formed particles as shown in Supplementary Fig. 2. The zeta potential value ranging from + 6.0 to + 8.4 mV indicated moderate colloidal stability of the nanosuspension, thereby lyophilization of the formed polymeric nanoparticles suspension was performed using the freeze-drier to improve the stability for long-term storage. Previous studies on polymeric nanoparticles have evidenced lyophilization as an upper hand to improve the stability of the nanoparticles^44–46^. The positive surface charge of the nanoparticles confirmed the greater potential for its uptake through the negatively charged cellular surface^47^.

The real-time stability data of the nanoparticles showed no significant changes in the drug entrapment efficiency (89.5% ± 0.5%), average particle size range (295 nm), PDI (0.230) and zeta potential (+ 7.57 mV) at the end of 6 months, which indicated the optimum stability of the formulation (Table 2 and Supplementary Fig. 3).

### In vitro drug release profile of 5-NPPP polymeric nanoparticles

**Figure 3:**
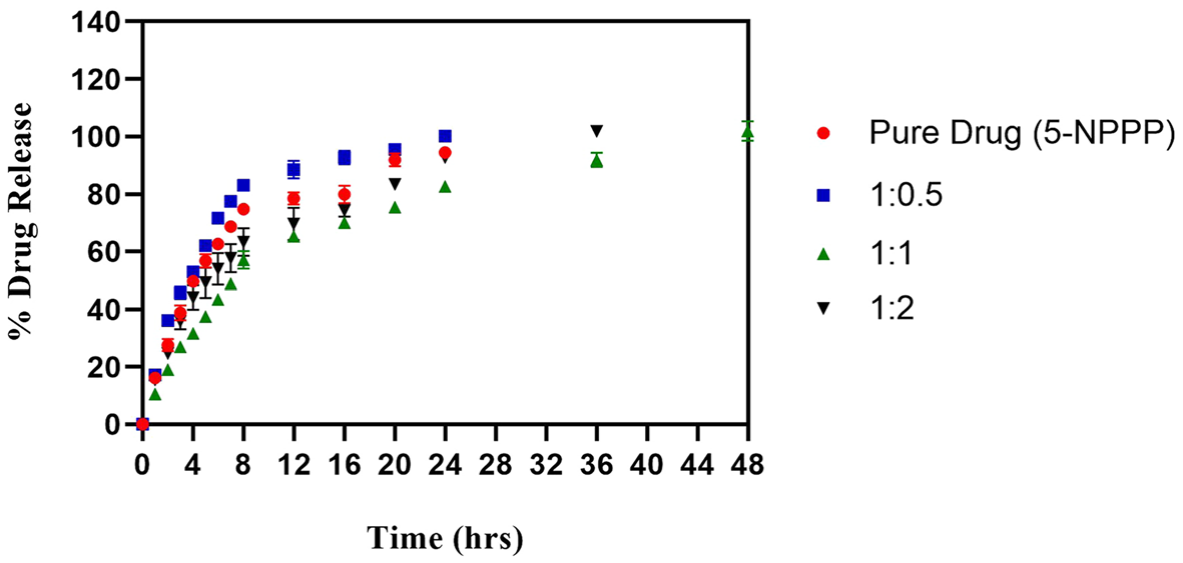
In vitro comparative drug release profile of pure 5-NPPP and 5-NPPP nanoparticles from varying ratios of the drug:polymer composition for a period of 50 h (n = 3, p < 0.05).

To modify the drug release pattern of 5-NPPP through polymeric nanoparticle preparation, Eudragit RSPO containing hydrophobic copolymer chains of poly-methyl methacrylate was selected as the sustained release polymer^48^. The release profile of 5-NPPP polymeric nanoparticles prepared with varying ratios of the drug:polymer (1:0.5, 1:1, and 1:2) ratio was studied by dialysis bag method and compared to the release pattern of pure 5-NPPP solution. For the pure sample of the 5-NPPP solution, about 80% of the drug was released out of the dialysis membrane at 12 h, followed by complete release overnight to attain the maximum 100% at the end of 24 h. In the case of the nanoparticles, the formulation containing 1:0.5 ratio drug: polymer showed a burst release of 36% at 2 h, followed by 80% release at 8 h that reached a cumulative maximum of 100% at the end of 24 h, which was like the pure drug release profile. When the composition of nanoparticles was varied between as 1:1 and 1:2 ratio, the drug release was retarded with a burst release of around 20% at 1 h, increasing to 60–70% at the end of 8 h, followed by a linear increase in the drug release to reach 100% at 36–48 h as reported (Fig. 3). The burst release (within 1 h) of 10–15% of the drug from the nanoparticle’s suspension could be ascribed to the unentrapped drug molecules and drug present on the surface of particles. Based on the lowest particle size, uniform monodisperse nature, optimum colloidal stability, maximum entrapment efficiency, and linear sustained release for a prolonged time, the nanoparticles developed with a 1:1 ratio of 5-NPPP and Eudragit RSPO polymer were selected for loading in the PVA hydrogel matrix.

Similar results have been demonstrated by Eudragit RSPO polymeric nanoparticles loaded with an oral anti-hyperglycemic agent Alogliptin, wherein the prepared nanoparticles exhibited an average particle size of 272 nm, the highest entrapment of 95% and prolonged release profile reaching 84% at the end of 24 h^49^. In another study, dual controlled release nanoparticles of Betaxolol hydrochloride was developed using montmorillonite mineral and Eudragit PO polymer, wherein an initial burst release of surface-bound drug was observed, followed by a slow release due to slow erosion, and maximum drug release of 80% by the complete breakdown of particles after 10 h^50^.

### Scanning electron microscopy analysis of 5-NPPP polymeric nanoparticles

**Figure 4:**
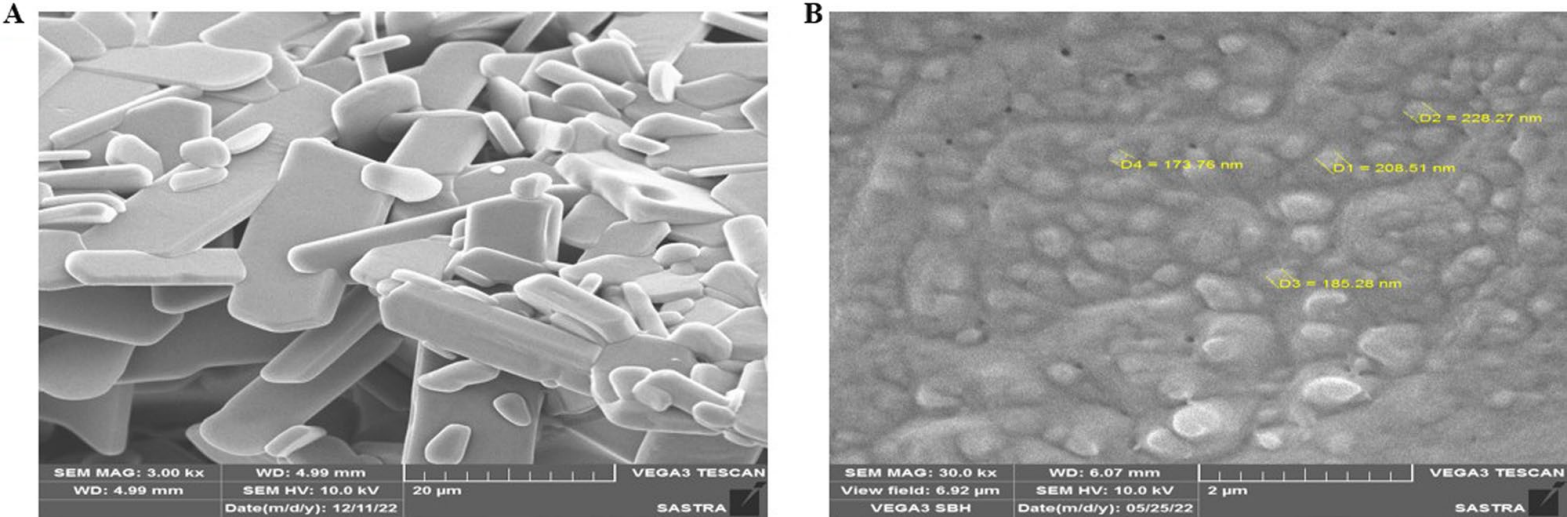
Scanning electron microscopic analysis of (**A**) Pure 5-NPPP and (**B**) Polymeric nanoparticles of 5-NPPP. (Magnification: 3.00 kx and 30.00 kx, respectively).

The scanning electron microscopic images of pure 5-NPPP exhibited irregular flakes of particles, whereas the 5-NPPP polymeric nanoparticles displayed clusters of nano-sized particles (Fig. 4A,B). The aggregation of the nanoparticles could be during the lyophilization process of the suspension that was stabilized by the addition of surfactant by the nanoprecipitation technique^47^.

### X-ray diffraction analysis (XRD) of 5-NPPP polymeric nanoparticles

**Figure 5:**
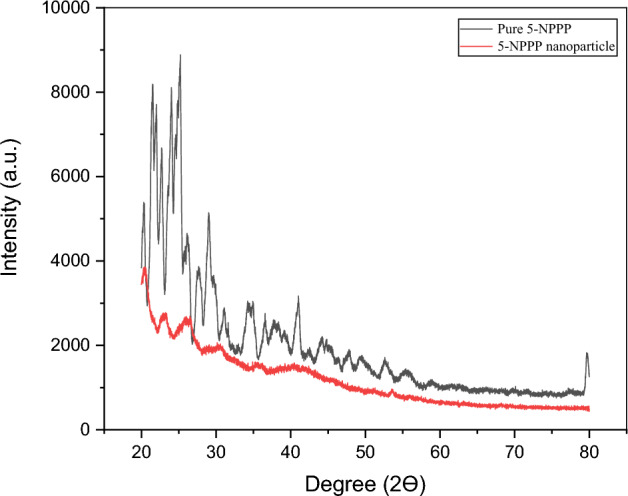
XRD analysis of pure 5-NPPP and 5-NPPP nanoparticles. Black peak denotes the pure 5-NPPP and red peak denotes polymeric nanoparticles.

The results of the XRD analysis of the pure 5-NPPP drug and the lyophilized sample of 5-NPPP polymeric nanoparticles are shown in (Fig. 5). The diffraction pattern of pure 5-NPPP indicated the characteristic sharp and intense peaks at ~ 20.575°, 23.267°, 26.507°, 53.583° and 55.924°. The diffractogram of the nanoparticles displayed clusters of broad peaks in the same range for the 2-theta degree measurements. However, the intensity of the peaks was reduced and scattered, indicating the drug’s transition from crystalline to semi-crystalline or amorphous state in the nanoparticles due to the encapsulation of the drug within the polymer matrix^51^.

### TGA–DSC analysis of 5-NPPP polymeric nanoparticles

**Figure 6:**
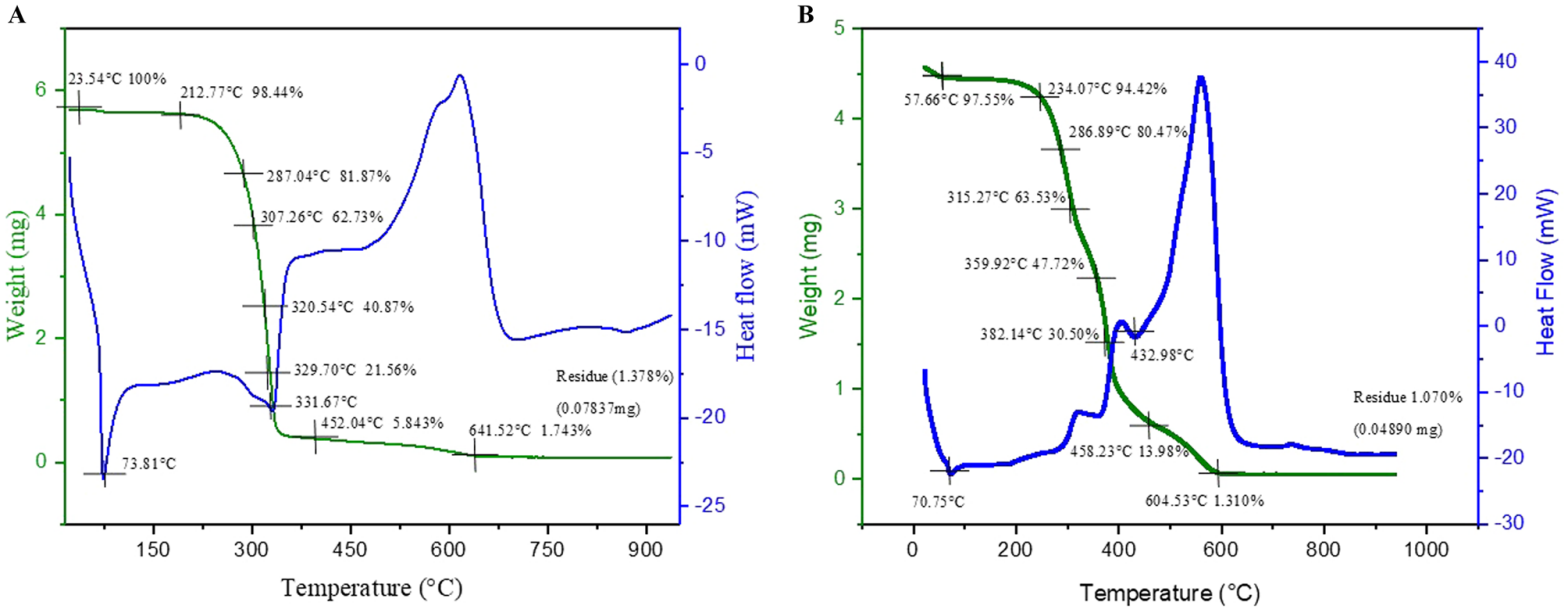
TGA–DSC comparative analysis of (**A**) Pure 5-NPPP and (**B**) 5-NPPP polymeric nanoparticles. TGA–DSC analysis of the pure 5-NPPP drug. The representation showed the finding of the melting point of novel scaffold (**A**) at 73.81 °C and degradation at 331.67 °C, further, they also showed a faster degradation state revealing the unstable nature of the scaffold when compared to the stable nature of polymeric nanoparticles (**B**). Green line indicate TG curve and blue line indicates DSC curve.

The TGA–DSC analysis was used to characterize the thermal behavior of the synthesized pure sample of 5-NPPP (Fig. 6A). The DSC thermogram displayed the critical melting point of the drug at 73.81 °C by the sharp endothermic peak. Further increase in the temperature showed another endothermic peak at 331.67 °C due to the degradation of the sample. Correspondingly, a sharp decline in the sample weight was observed from 100 to < 10% in the TGA curve. Further, the drug degradation was slow from 350 to 600 °C and completed with an exothermic peak in the DSC curve, leading to the residual weight of 1.378% in the TG curve.

In the case of 5-NPPP polymeric nanoparticles, the melting point of the drug was identified at 70.75 °C in the DSC thermogram, which was slightly shifted compared to the pure sample. Also, the endothermic peak was not sharp since the crystallinity of the drug was diminished due to its entrapment within the amorphous polymer matrix. Further analysis showed an insight into the improved stability of the nanoparticle’s formulation across 300–400 °C. Accordingly, the TG curve displayed a gradual decrement in weight loss due to step-wise degradation of the sample followed by complete decomposition at 600 °C with a residual weight of 1.070%. Overall, the purity and stability of the drug were not affected by entrapping in the polymeric nanoparticle formulation^51^ (Fig. 6B).

### FTIR analysis of 5-NPPP polymeric nanoparticles

**Figure 7:**
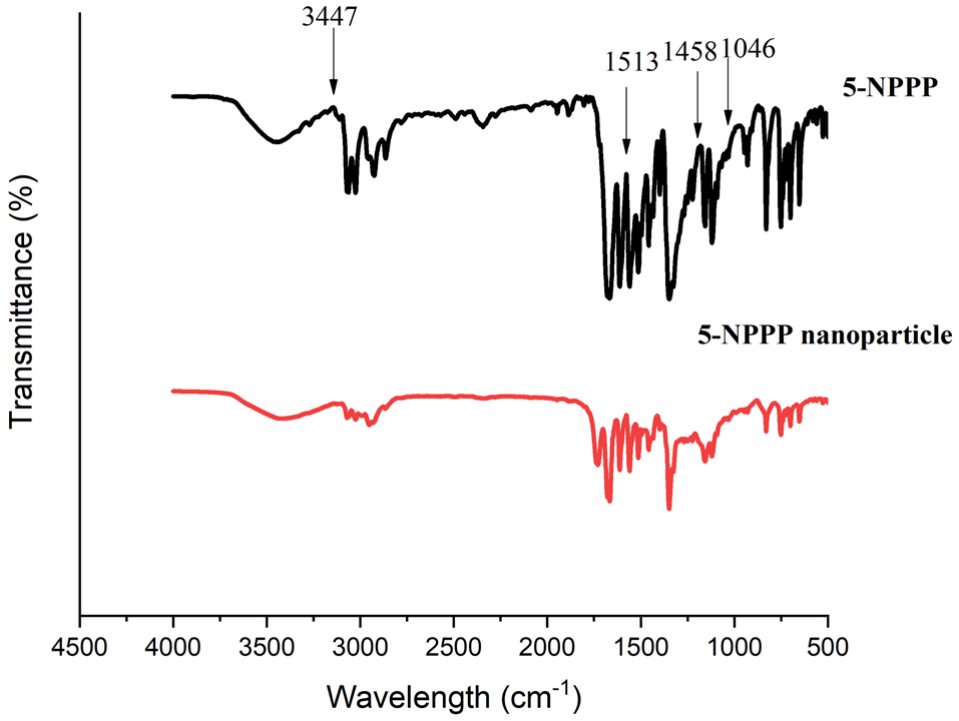
FTIR spectra of 5-NPPP polymeric nanoparticles compared to pure 5-NPPP.

The FTIR spectrum of the pure sample of 5-NPPP exhibited peaks at 1513 cm^−1^, 3447 cm^−1^, 1458 cm^−1^, and 1046 cm^−1^ indicating the vibrational stretching modes of –N–O, –N–H, –CH_3,_ and C–O, respectively. In the FTIR spectrum of the lyophilized 5-NPPP polymeric nanoparticles, although certain peaks of the drug and polymer were overlapped and slightly shifted, the characteristic identity peaks of the 5-NPPP drug were observed at 1513 cm^−1^ for –N–O stretching and 3447 cm^−1^ for the presence –N–H functional group. Thus, it could be concluded that the 5-NPPP drug molecules were stable and entrapped within the polymeric matrix (Fig. 7) as similar findings were found in previous research work^52^.

### FTIR analysis of hydrogels

**Figure 8:**
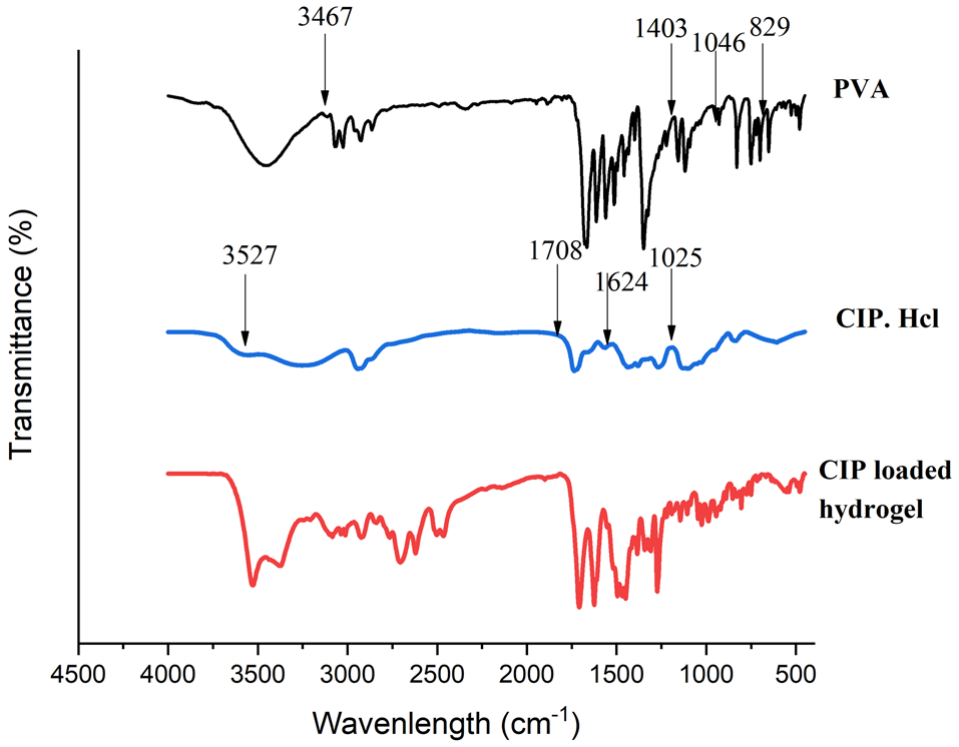
FTIR spectra of Ciprofloxacin hydrochloride loaded hydrogel compared to pure Ciprofloxacin hydrochloride. The resemblance of similar peaks confirms the entrapment of the drug within the pockets of the hydrogel matrix.

The FTIR analysis of CIP showed the identity peaks of the characteristic functional groups at 3527 cm^−1^ for O–H stretching, 1708 cm^−1^ for C=O stretching, 1025 cm^−1^ for the C–F group, and 1624 cm^−1^ quinolone group, respectively. The FTIR analysis of polymer PVA exhibited characteristic peaks at 3467 cm^−1^, 1403 cm^−1^, 1046 cm^−1^, and 829 cm^−1^, displaying the vibrational modes of O–H stretching, –CH_2_ bending, C–O stretching and C=C stretching, respectively. In the case of the FTIR spectrum of CIP-loaded hydrogel, the peaks of the drug and polymer were merged and overlapped due to mild interactions between the drug and polymer, as the drug was entrapped into the polymeric matrix during the crosslinking of hydrogels similar to the previous research works^53^. For example, the peak at 3527 cm^−1^ was diminished, possibly due to the hydrogen bonding interaction of the –OH groups. Although certain peaks were slightly shifted in the hydrogels compared to the pure drug, the specific peaks of the characteristic functional groups like C=O, C–F, and quinolone of the CIP molecule remained the same, which confirmed the stability of the drug in the hydrogel as shown in Fig. 8.

### Swelling studies of CIP and 5-NPPP nanoparticles hydrogel

**Figure 9:**
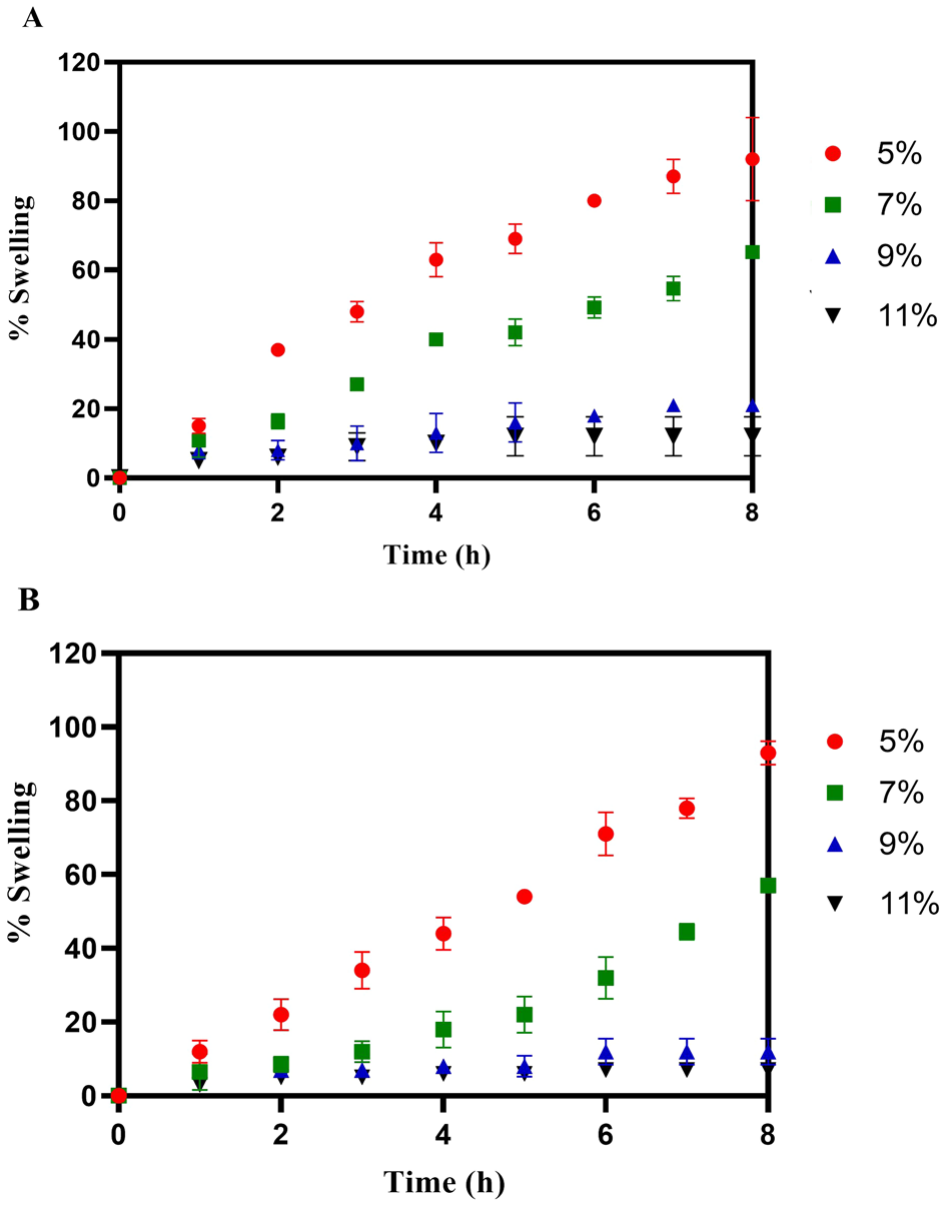
In vitro Swelling studies of (**A**) Ciprofloxacin hydrochloride loaded hydrogels and (**B**) 5-NPPP nanoparticle loaded hydrogels (n = 3, p < 0.05).

Swelling is one of the most important characteristic features considered to investigate the nature of the hydrogels, drug release, and mechanical stability, which depends on the cross-linked network structure, amount of crosslinking agent used, and hydrophilicity of the functional groups present in the polymer to gain insight into the absorption of the water molecules. The swelling pattern of the hydrogels showed that the swelling was higher at lower polymer concentrations due to the limited crosslinking of the available polymer molecules in the matrix. Whereas, in the case of the higher percentage of polymer, the swelling rate was found to be lesser, which confirmed the increase in crosslinking between a more significant number of polymer molecules in the same volume matrix^54,55^. In the case of CIP-loaded hydrogels, when the concentration of PVA polymer was varied as 5%, 7%, 9%, and 11%, the swelling index was decreased to 92%, 65%, 21%, and 12%, respectively at the end of 8 h (Fig. 9A). A similar trend was observed in the 5-NPPP-loaded hydrogels, and the swelling index ranged from 95 to 40% as the polymer concentration changed from 5 to 11% proportionately. The rapid swelling had resulted in a faster release of 5-NPPP from the hydrogels, which necessitated the need for the development of sustained-release polymeric nanoparticles of the drug to be loaded into the hydrogels. The 5-NPPP nanoparticles loaded hydrogels displayed the swelling index of 93%, 57%, 12%, and 7% for the hydrogels containing 5–11% w/v PVA concentration, correspondingly (Fig. 9B). The higher reduction of a swelling index in the nanoparticles-loaded hydrogels, especially from 7 to 11% could be attributed to the interactions of the polymer present in the nanoparticles within the cross-linked hydrogel matrix. There was no significant difference in the swelling behavior of the hydrogels in phosphate buffer pH 7.4 and 5.8. As the degree of swelling was less with increased polymer concentration, it could lead to slow diffusion of drug molecules/nanoparticles through the hydrogel matrix's swollen pores, resulting in sustained release of the drug^56–58^. Another study validated that when the concentration of polymer was decreased with a constant amount of crosslinking agent, the monomers had cross-linked with greater levels of non-uniform spacing, revealing higher rates of swelling and was contrary when polymer concentration was increased^59^.

### In vitro drug release profile of 5-NPPP nanoparticles-loaded hydrogels

**Figure 10:**
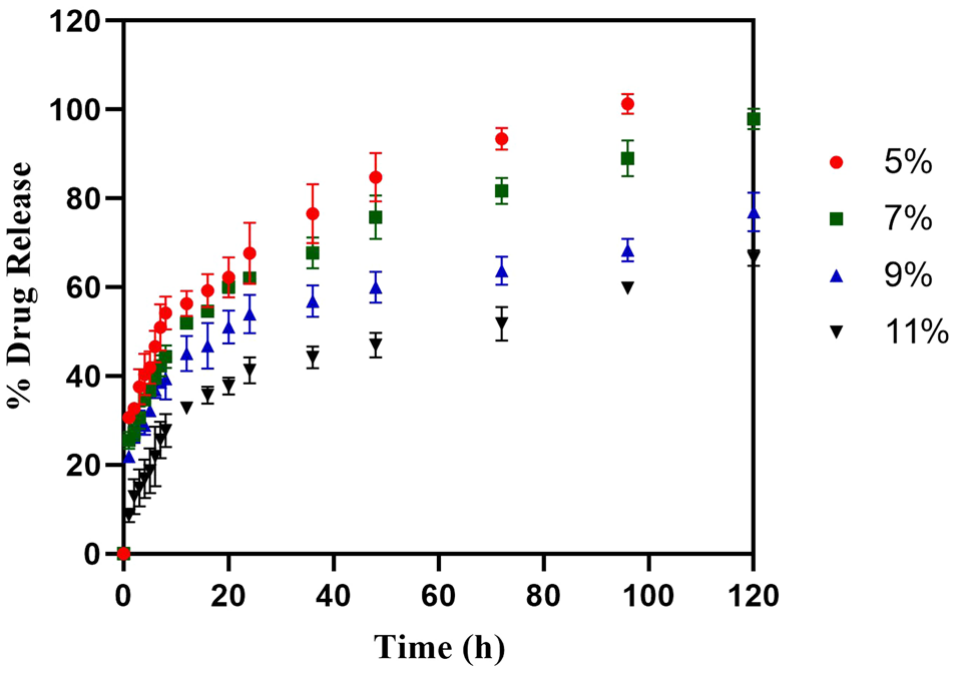
Cumulative (%) release of 5-NPPP nanoparticles loaded in varying percentages of PVA and was studied for a time period of 5 days (n = 3, p < 0.05).

The optimized 5-NPPP polymeric nanoparticles loaded hydrogels with 5–11% PVA had demonstrated more sustained release behavior for the period of 4–5 days in phosphate buffer pH 7.4 and 5.8, compared to the 5-NPPP nanoparticles and the hydrogels alone (around 2 days only). The cumulative drug release from the hydrogels prepared with 5%, 7%, 9%, and 11% w/v PVA were 100% (96 h), 100% (120 h), 77% (120 h), and 66% (120 h), respectively (Fig. 10). The results proved that the hydrogels with a lower concentration of PVA (5% w/v) could provide sustained release for up to 4 days, and, a further increase in the concentration (7–11% w/v) could retard the drug release to ≥ 5 days. This could be attributed to the dual barrier release effect caused by the presence of the pH-independent sustained release polymers, Eudragit RSPO in the matrix nanoparticles, and the cross-linked PVA hydrogel system^60^.

Based on the maximum cumulative concentration reached at the end of 5 days, the 7% w/v PVA hydrogels were selected as an optimum component matrix for delivering both drugs. The hydrogels loaded with both CIP (free drug) and 5-NPPP polymeric nanoparticles displayed an analogous drug release profile as the individual drug-loaded hydrogels. Based on the in vitro research findings, the optimized hydrogels (7% w/v PVA) loaded with CIP and 5-NPPP nanoparticles that provided a sustained release for a period of 5 days were considered for the in vivo studies in mice models infected with *S. aureus* soft skin infections.

### Drug release kinetics

The kinetics of drug release from the nanoparticles and the hydrogel formulations were studied by fitting the in vitro drug release data into various mathematical models. The selected 5-NPPP polymeric nanoparticles were best fitted to Weibull (R^2^ = 0.9943) and Makoid–Banakar (R^2^ = 0.9911) models, which explained the drug release from matrix type of nanoparticles and influenced by the type and composition of the polymer, respectively. In the case of the hydrogels loaded with CIP and 5-NPPP nanoparticles individually, the highest correlation (R^2^) was found with Makoid Banakar (R^2^ = 0.9941 and 0.9960) and Korsemeyer–Peppas models (R^2^ = 0.9967 and 0.9965), which explained the drug release based on the diffusion mechanism that was predominantly influenced by the polymers. Additionally, the n-value (< 0.45) of 0.303, 0.293, and 0.441 for CIP-loaded hydrogels, 5-NPPP nanoparticles hydrogels, and the 5-NPPP polymeric nanoparticles, respectively had explained the diffusion of drug following Fick’s law based on concentration gradient as the driving force. Since perfect sink conditions were maintained in the drug release experiments, a maximum concentration gradient was achieved to mimic the in vivo systemic circulation conditions (Supplementary Table 1).

### In vitro diffusion study of the hydrogels loaded with ciprofloxacin and 5-NPPP nano-particles

**Figure 11:**
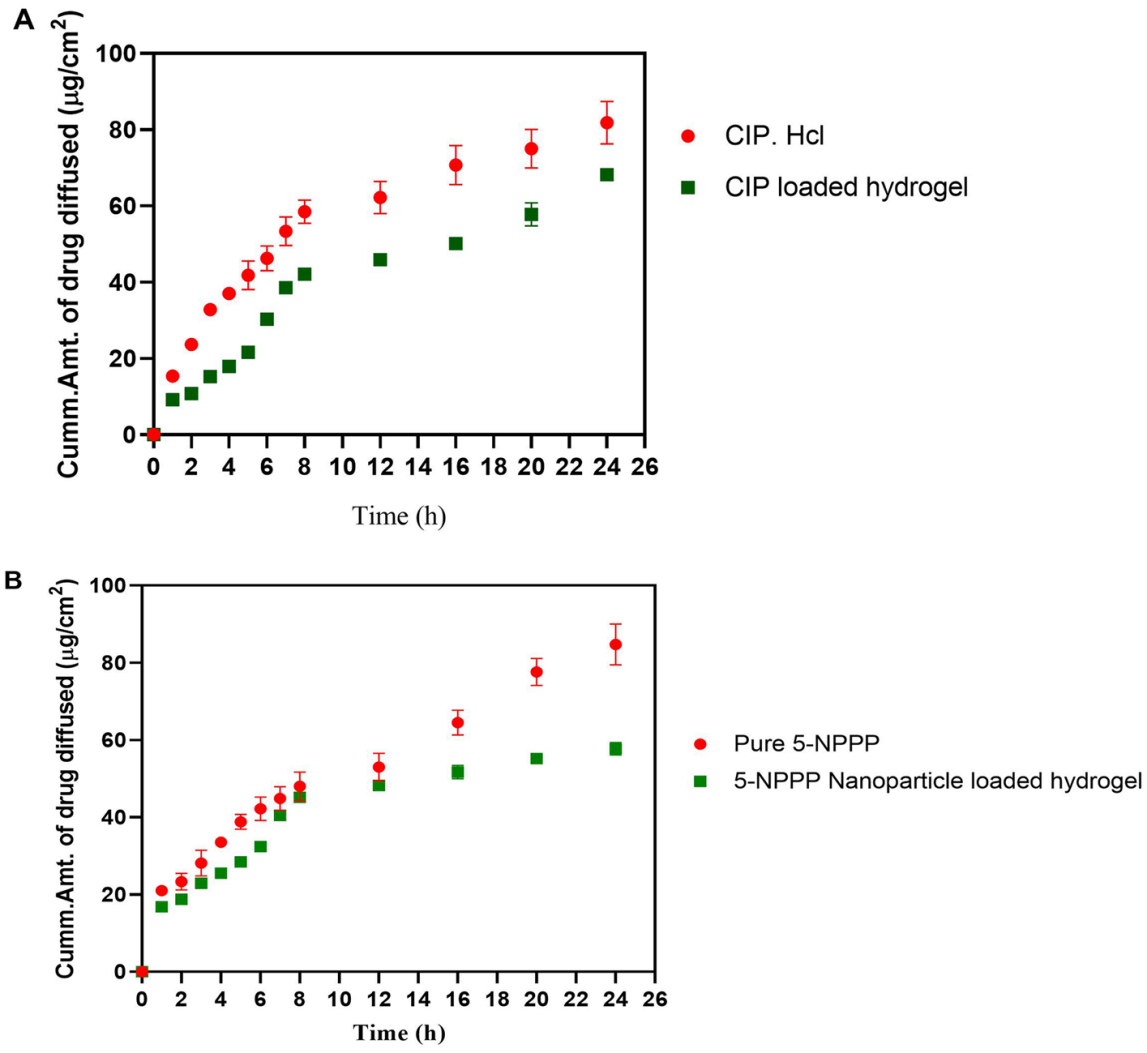
In vitro permeation analysis of drug from drug-loaded hydrogels in comparison with the permeation of pure drugs. (**A**) Comparative permeation study of ciprofloxacin from ciprofloxacin hydrochloride vs pure ciprofloxacin hydrochloride; (**B**) Comparative permeation of 5-NPPP nanoparticle from 5-NPPP nanoparticle loaded hydrogel vs pure 5-NPPP (n = 3, p < 0.05).

The in vitro diffusion was studied for the hydrogels loaded with CIP and 5-NPPP nanoparticles individually, using a cellulose acetate membrane for a time period of 24 h. The hydrogels exhibited lesser diffusion of the molecules across the membrane compared to the trend shown by respective pure drugs, which could be due to the slow release from the matrix. In the case of CIP-loaded hydrogels, the Q_24_ (Quantity of material passing through the membrane at 24 h) was found to be 68.21 ± 0.13 µg/cm^2^, which was reduced compared to the pure CIP 81.82 ± 5.57 µg/cm^2^. In a similar trend, the Q_24_ for the 5-NPPP and its nanoparticles-loaded hydrogels was found to be 84.73 ± 5.27 µg/cm^2^ and 69.36 ± 1.48 µg/cm^2^, respectively. The trend was comparable to the in vitro drug release profiles, which confirmed that the drug released from the hydrogel dosage forms could proportionately diffuse through biological membranes in vivo. The diffusion of the NPPP molecules (efflux pump inhibitor) was similar to the CIP molecules, which would be advantageous for the enhanced adjuvant effect, thereby improving the therapeutic effect of the antibiotic at reduced doses, as shown in (Fig. 11A,B).

### Acute dermal toxicity

**Figure 12:**
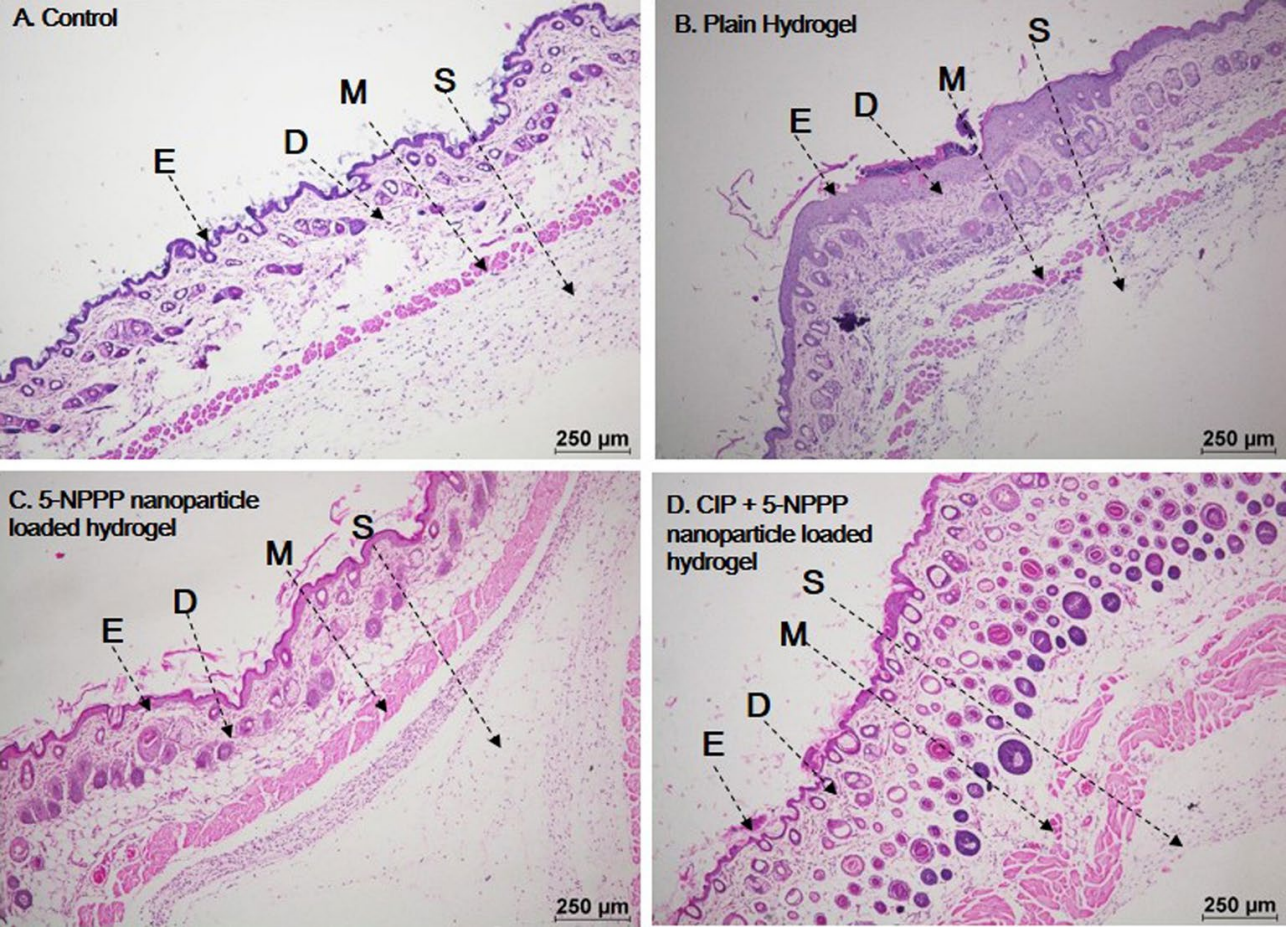
Histopathological analysis of skin taken for the acute dermal toxicity analysis. The hydrogels w/o the drugs were found to be safer and non-toxic showing no adverse effects. *E* epidermis, *D* dermis, *M* muscularis, *S* subcutis.

The female Balb/c mice were chosen in the prescribed age category as per the OECD guidelines^61^. The weight of the animals was measured before the hair was trimmed. The hydrogels were placed on the skin and were bandaged and were observed twice daily for regular motor activity. Animal weights were taken on the seventh day after the removal of hydrogels (if any were damaged) and new hydrogels were again replaced on the same day and weight was taken on the fourteenth day. All the mice were sacrificed by euthanasia on the fourteenth day and skin samples were collected for histopathological analysis. No allergic reactions, convulsions, salivation, or diarrhoea, were observed. Mice were found to be active and no ulcer formation or wound formation was observed on the skin. Promisingly the hydrogels with drugs were found to be safer and non-toxic as shown in Fig. 12. Interestingly further investigation showed no systemic or abnormal issues in the mice taken for the study. Feed intake of the mice were found to be at normal levels and no mortality was reported as shown in Supplementary Tables 3 and 4.

### In vivo drug efficacy

**Figure 13:**
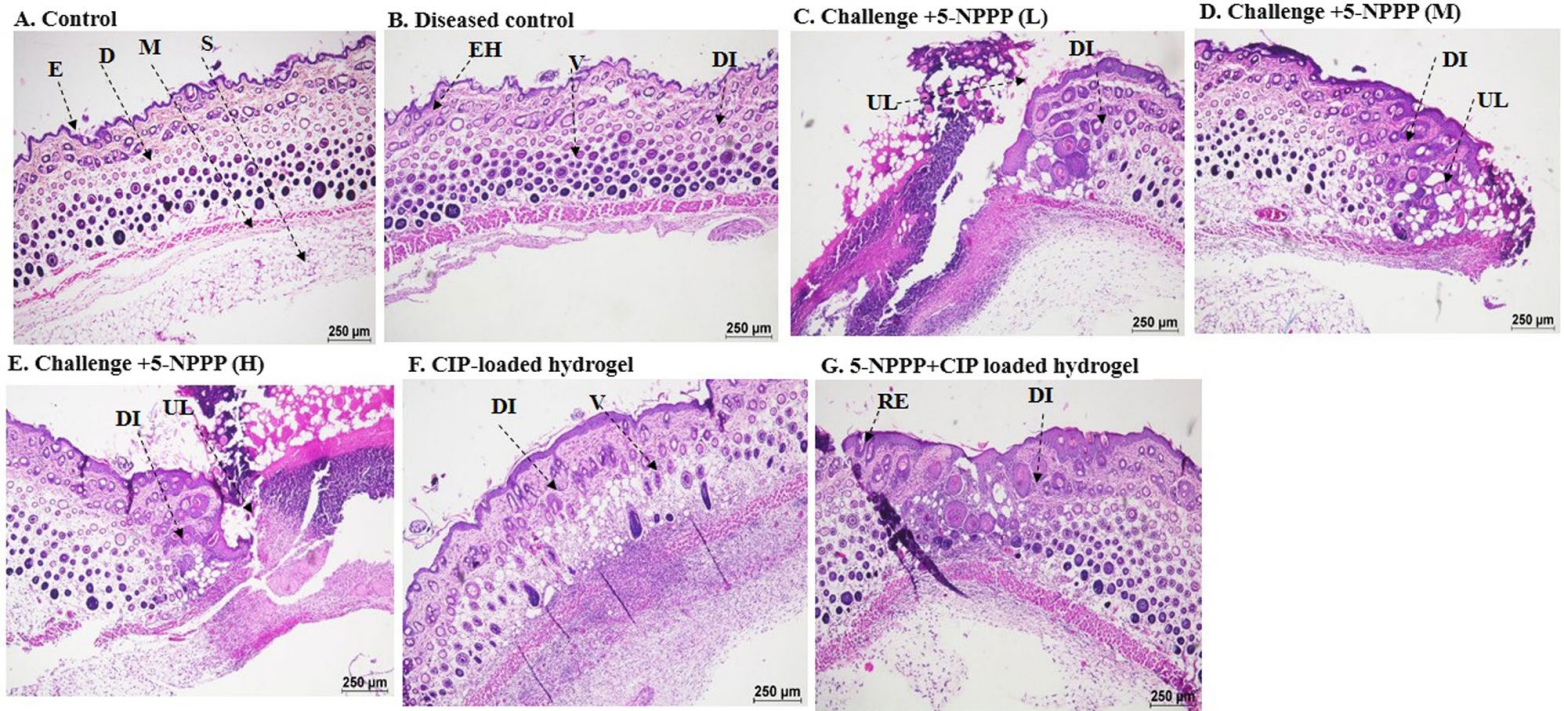
Histopathological analysis of skin taken for investigating the drug efficacy analysis against infections. The hydrogels w/o the drugs were found to be safer and non-toxic showing no adverse effects.*E* epidermal, *D* dermal, *M* muscularis, *S* subcutis, *DI* dermal inflammation, *EH* epidermal hyperplasia, *V* vacuolation, *UL* ulcer and *RE* re-epithelisation. There were no major abnormal findings, the findings were found to be minimal and negligible.

In order to exert a therapeutic effect, the drugs will be evaluated for their effectiveness and efficacy in treating infections. The formation of the infection was noticed after 24 h of the infection, as the treatment was given in the primary phase of the infection. The results showed that medium dosage of the dual drug-loaded hydrogels were safer in treating the infections, further, observation showed medium dosage loaded hydrogels were optimum in treating the infection producing a synergistic effect. The dual drug-loaded hydrogels showed the events of re-epithelization, whereas the CIP-loaded hydrogels showed the events of infection that were similar to the diseased control. The infection rate was found to be higher in mice treated with CIP-loaded hydrogels. The weights of the spleen in were measured at the end of the study, and an increment in the weight and length was observed in the infected control group which is due to the inflammation that occurred during the infection as shown in Tables 3 and 4. Further, the weights of the animals were compared before the induction and before the necropsy was performed, it was noted that the weight of the animals was reduced in the infected control group, whereas in the treatment group weight loss was not observed due to the clearance of the infection as shown in Fig. 13. The current study was an initiative step in exploring and utilizing a novel scaffold as an efflux-pump inhibitor and as an adjuvant promisingly potentiating and demonstrating synergistic interaction with antibiotics and being a promising therapy to treat soft skin infections as evidenced by in vivo experiments.

**Table 3:** Spleen weight for the male Balb/c mice (n = 3).

Group	Average weight of spleen (g)	Std. dev.
Control male (shave)	0.125	0.029
Disease control (male)	0.230	0.006
Treatment 1—Low (5-NPPP) (male)	0.069	0.017
Treatment 1—Medium (5-NPPP) (male)	0.185	0.049
Treatment 1—High (5-NPPP) (male)	0.154	0.065
Treatment—CPX (male)	0.247	0.012
Treatment—CPX + 5-NPPP (male)	0.125	0.029

**Table 4:** Spleen weight for the female Balb/c mice (n = 3).

Group	Average weight of spleen (g)	Std. dev.
Control female (shave)	0.175	0.004
Disease control (female)	0.232	0.076
Treatment 1—Low (5-NPPP) (female)	0.161	0.062
Treatment 1—Medium (5-NPPP) (female)	0.159	0.018
Treatment 1—High (5-NPPP) (female)	0.199	0.074
Treatment—CPX (female)	0.298	0.011
Treatment—CPX + 5-NPPP (female)	0.183	0.047

### In vivo serial passage assay

**Figure 14:**
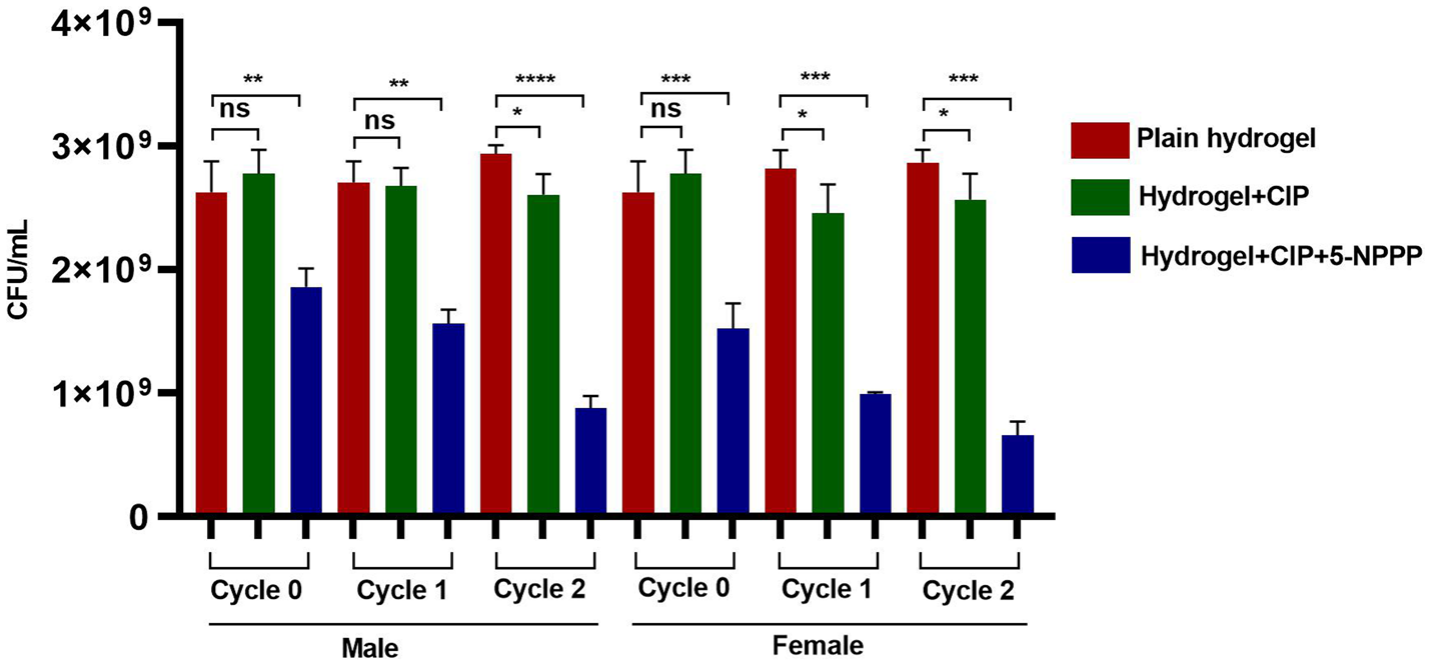
In vivo drug resistance passage evaluation in both genders of mice upon treatment with CIP and dual combination loaded hydrogels. Control is being considered as a plain hydrogel. Mice were infected with *S. aureus* 1199B and were treated to evaluate the drug resistance pattern. Unpaired student test, *p < 0.05, **p < 0.01, ***p < 0.0001.

Although the treatment regimens are efficient, prolonged exposure and inappropriate antibiotic usage may lead to treatment failure in the host-infected models. Hence the therapeutic formulations (5-NPPP (0.5 µg/mL) and CIP (0.5 µg/mL)) were tested for tolerance/resistance induction to SA-1199B (CIP-resistant; CIP^R^) infected mice. In parallel, the other treatment groups (CIP, plain hydrogel) were used for a comparative assessment. The serial passage experiment for all the groups was carried out for two cycles (cycle 0,1,2) at 24 h intervals. There was no significant difference in bacterial load when treated with plain hydrogel and CIP, which clearly indicates that SA-1199B has a strong resistance to CIP. However, the combinatorial treatment (5-NPPP and CIP) significantly reduced CFU/mL. This corroborates with the previously reported data that 5-NPPP in combination with CIP exhibits an increased activity with a 16-fold reduction in concentration CIP^25^, as shown in Fig. 14.

## Materials and methods

### Materials

The pure drug sample Ciprofloxacin Hcl (CIP) was purchased from Sigma Aldrich, and the compound 5-NPPP was synthesized in-house, at SASTRA Deemed University, India. Poly Vinyl Alcohol (MW 146,000 g/mol, 99+% hydrolyzed) was procured from Sigma Aldrich, India. Chemicals such as glutaraldehyde, acetic acid, and acetone used in the study were analytical grade.

### Bacterial strains and growth conditions

The fluoroquinolone-resistant strain Sa-1199B was obtained from BEI Resources, USA. The strain was cultured in Muller Hinton Broth (MHB, Himedia, Mumbai, India) and was incubated at 37 °C for 24 h. The cultures were further streaked in Muller Hinton Agar plates and were used for the current study.

### Pre-formulation studies of the drugs

Solubility studies of the drugs CIP and 5-NPPP were performed using ethanol, methanol, acetone, dichloromethane, isopropyl alcohol, chloroform, dimethyl sulfoxide, distilled water, and phosphate buffers at different pH. The standard calibration of CIP and 5-NPPP was carried out using phosphate buffer solution pH 7.4 and pH 5.8 media for quantitative estimation of the drugs using a UV–Visible spectrophotometer based on Beer-Lambert’s law. The stock solution of 1 mg/mL concentration was prepared and serially diluted to obtain the solutions with concentrations from 5 to 25 µg/mL. By scanning the solutions in a UV–Visible spectrophotometer (UV-1800, Shimadzu, Japan) at the wavelength range of 200–800 nm, the λ_max_ of both the molecules was identified, which was fixed for analysis of all the samples. The calibration protocol was repeated thrice for both drugs to validate the analytical method. A standard calibration graph (Supplementary Fig. 4) was plotted between the known concentration of samples and the respective absorbance obtained, to check the correlation factor, linear regression, and equation of the straight line. This linear standard curve data was utilized for further analysis of drugs to estimate the drug content, entrapment efficiency, in vitro release, and ex vivo permeation.

### Optimization of crosslinking agent and catalyst for the preparation of hydrogels

Plain PVA hydrogels were prepared using various concentrations of polymer (5%, 7%, 9%, and 11% w/v). The specified weight of the polymer was dissolved in the required volume of distilled water by placing it on a thermostat hot plate magnetic stirrer with continuous stirring at 500 rpm. Further, bath sonication was performed to obtain a homogenous polymer dispersion without the formation of air bubbles. Hydrogels were prepared by pouring 1 mL volume of the polymer solution in a round-shaped mold of diameter 2.5 cm, wherein glutaraldehyde was added as a crosslinking agent and glacial acetic acid was added as a catalyst, each at different concentrations of 0.25%, 0.5%, and 1.0% v/v. The molds were placed at room temperature until the completion of crosslinking to obtain an insoluble hydrogel matrix. The air-dried hydrogels were evaluated for dimensions, weight, integrity, and swelling index, based on that 0.5% v/v glutaraldehyde and 0.25% v/v glacial acetic acid was selected to be optimum for the hydrogels, which was reserved as control blank hydrogel samples.

### Preparation of CIP and 5-NPPP-loaded hydrogels

The hydrogel gel forming PVA solutions were subjected to rheology analysis to check the viscosity using Brookfield Viscometer (LVDV-II + Pro, Brookfield Engineering, USA) with spindle no. 64. The increase in shear rate from 30 to 150 rpm had shown a slight decrease in the viscosity from 140 to 123 cP for the 7% PVA hydrogel forming solution (Supplementary Fig. 5), which confirmed the non-Newtonian flow with slight shear-thinning property suitable for polymeric cross-linking application^62^.

To the prepared PVA polymer solution at various concentrations, a precisely weighed amount of CIP was added and mixed thoroughly to obtain the uniform drug concentration of 1 mg/mL. About 1 mL of the mixture was poured into the mold, followed by adding 0.5% glutaraldehyde and 0.25% glacial acetic acid as chemical cross-linker and catalysts, respectively. The molds were incubated at room temperature until the completion of cross-linking to obtain each hydrogel with a 1 mg dose of the drug. A similar protocol was trialed for the compound 5-NPPP-loaded hydrogels.

### Preparation of polymeric nanoparticles of 5-NPPP

The formulation of polymeric nanoparticles of 5-NPPP was carried out by modified nanoprecipitation—solvent evaporation technique using Eudragit RSPO polymer at a varying ratio in addition to PVA as a surfactant for aiding in the formation of stable nanoparticles. About 10 mL of 0.5% w/v surfactant solution was prepared as the aqueous phase. The required amount of the 5-NPPP and polymer Eudragit RSPO at the ratio of 1:0.5, 1:1, and 1:2 was taken and dissolved in 1–2 mL of acetone to form the non-aqueous phase. The non-aqueous phase was mixed thoroughly and loaded into a syringe, and then added drop-wise to the aqueous PVA solution with continuous stirring for > 2 h, to allow nanoprecipitation due to evaporation of the organic solvent followed by the formation and stabilization of polymeric nanoparticles of 5-NPPP^63–65^. After the complete removal of organic solvent, the obtained nanosuspension was stored in a refrigerated condition, which was further lyophilized (Christ Alpha 2-4 LD Plus, Osterode Am Harz, Germany) for 48 h to obtain freeze-dried nanoparticles for the analytical characterization studies. The real-time stability study was performed for the selected nanoparticle formulation stored at 30 °C/65%RH as per the ICH guidelines for 6 months and evaluated for the drug entrapment efficiency and particle size behaviour on storage.

### Entrapment efficiency of 5-NPPP polymeric nanoparticles

The amount of 5-NPPP entrapped within the polymeric nanoparticles was quantified by calculating the concentration of the drug in the pellet (entrapped molecules) and supernatant solution (unentrapped soluble molecules), obtained after centrifugation of 1 mL of the nanoparticles suspension at 10,000 rpm for 15 min. The supernatant and pellet were collected and dissolved separately using 10 mL of ethanol and the absorbance of the solutions was noted at 304.5 nm using a UV–Visible spectrophotometer with the respective buffer as the blank solution.

The entrapment efficiency was found using the formula

$$\% Entrapment\;Efficiency = \frac{Total\;drug\;content - Drug\;content\;in\;supernatant}{{Total\;drug\;content}} \times 100$$

### Particle and zeta potential of 5-NPPP polymeric nanoparticles

The particle size distribution and surface charge of the formed 5-NPPP polymeric nanoparticles were determined using Zeta Sizer (Malvern Instruments, UK). About 0.5 mL of the nanoparticles suspension was diluted with water, placed in the sample cuvette, and analyzed using dynamic light scattering and conductivity principles.

### In vitro release studies of polymeric nanoparticles

The in vitro release studies of 5-NPPP polymeric nanoparticles were performed by the dialysis bag method. About 0.5 mL of nanosuspension was taken in a dialysis membrane bag and immersed into a vial containing 5 mL of phosphate buffer and placed in a magnetic stirrer at 100 rpm, to maintain uniform contact of the bag with the buffer. At regular intervals of a period, 5 mL of the solution was withdrawn and replaced with an equal volume of fresh buffer to maintain perfect sink condition. The absorbance of the collected samples was quantified using UV–Visible spectroscopy at 304.5 nm and concentration was quantified using standard calibration data.

### Analytical characterization of the 5-NPPP polymeric nanoparticles

#### Scanning electron microscopy characterization

The surface morphology of 5-NPPP nanoparticles was examined by scanning electron microscopic technique (VEGA3, TESCAN, Czech Republic) and compared to the pure sample. The samples were taken on the stub using a thin brush and gold sputter-coated (Sputter coater, JEOL, Japan) to form a thin film of gold on the nanoparticles. The coated sample was placed inside the test chamber and the images were captured at the accelerating voltage of 3 kV and magnification of 10,000×–40,000×^66^.

#### X-ray diffraction analysis

Solid-state characterization of 5-NPPP and its polymeric nanoparticles was performed by XRD using a Bruker-D8 focus diffractometer. The technique involved the exposure of samples to nickel-filtered CuKα radiation (40 kV, 40 mA, λ = 0.154 nm) and scanning from 20° to 80°, at 2Ɵ. The nature of lyophilized samples of 5-NPPP nanoparticles was compared to the pure 5-NPPP.

#### TGA–DSC characterization

The TGA/DSC analysis was performed for the 5-NPPP nanoparticles and compared to the pure drug sample to understand the thermal behavior and transition stages. The experiment was conducted using SDTQ600, TA instruments with the initial sample mass of 15 mg by heating to 220–950 °C min^−1^ in a dry air atmosphere with a flow of nitrogen at 20 bar.

#### Preparation of 5-NPPP polymeric nanoparticle loaded hydrogels

Based on the minimum particle size, monodisperse distribution, maximum stability, and higher entrapment efficiency, the optimized nanoparticles were selected for loading into the PVA hydrogels. The 5-NPPP polymeric nanoparticles incorporated hydrogels were prepared by a similar protocol, wherein the nanoparticles equivalent to the required amount of 5-NPPP were uniformly dispersed in the hydrogel matrix of PVA (7%, 9%, 11%, and 13% w/v) to obtain each hydrogel with 1 mg dose equivalent of the 5-NPPP compound.

#### Estimation of drug loading in the hydrogels

The hydrogels (n = 3) loaded with CIP drug/5-NPPP polymeric nanoparticles were placed in 5 mL of phosphate buffer individually and left overnight. After 24 h, they were crushed separately in addition to 5 mL of ethanol using a mortar and pestle^67–69^. The obtained solutions were filtered and diluted to 100 mL using phosphate buffer and the absorbance was measured at 271.5 nm and 304.5 nm for CIP and 5-NPPP, respectively. The concentration of drugs in individual hydrogels was calculated based on the standard calibration curve of the pure drugs. The amount of drug loading in the hydrogels was estimated by the formula,

$$\% \;Drug\;Loading = \frac{Drug\;content\;in\;crushed\;sample}{{Initial\;total\;drug\;content}} \times 100$$

#### Drug–polymer interaction studies of the nanoparticles and hydrogels by FTIR analysis

The presence of drugs and their interactions with the polymers were studied by FTIR spectra characterization using the instrument Spectrum 100 (Perkin Elmer). The dried and crushed hydrogels were mixed with potassium bromide in the proportion of 1:20 to form a thin transparent pellet by applying hydraulic pressure of 10t/cm^2^ pressure in 20 mbar vacuum, which was scanned in the range of 4000 to 400 cm^−1^ at the resolution of 4 cm^−1^. The selected formulations of 5-NPPP polymeric nanoparticles, the nanoparticles-loaded hydrogel, and the CIP-loaded hydrogel were compared to the respective pure drugs.

#### Swelling studies of the hydrogels

The formulated hydrogels were circular with an average diameter of 2.2 ± 0.05 cm and thickness of 0.5–0.6 mm. Swelling studies were performed for the hydrogels loaded with CIP, pure 5-NPPP molecules, and 5-NPPP nanoparticles individually. The initial weight of the hydrogels (n = 3) was noted and then, placed in a petri dish containing 10 mL of phosphate buffer. The hydrogels were then removed from the petri dish at regular intervals of time, tapped on tissue paper to drain the surface water, and observed for the weight. The difference between the initial and changing weights of the hydrogels was considered and the swelling index was calculated using the given formula and graphically represented by plotting between time on the x-axis vs. % swelling index on the y-axis^54^.

$$\% \;Swelling\;Index = \frac{Final\;Weight - Initial\;Weight}{{Initial\;Weight}} \times 100$$

#### In vitro drug release studies of hydrogels

The in vitro release studied of hydrogels loaded with CIP, 5-NPPP, and 5-NPPP nanoparticles was performed individually. The release study was conducted in phosphate buffer pH 5.8 and pH 7.4 to mimic the external surface skin pH and internal systemic pH, respectively^17,36,70^. The hydrogels (n = 3) were studied by using the USP type-2 dissolution apparatus with a paddle (Lab India, Mumbai, India) and the bowl containing 100 mL of media. The study was conducted by maintaining the outer bath temperature at 37 °C and the speed of the rotation at 50 rpm. About 5 mL of the sample was withdrawn from the bowl and replaced with an equal volume of fresh buffer solution, at appropriate time intervals for up to 5 days. The quantification of drug release was performed using UV–Visible spectrophotometer by measuring the absorbance of CIP at 271.5 nm and 5-NPPP at 304.5 nm. The data are reported as the mean ± standard deviation (n = 3)^54^.

#### Release kinetics analysis

To understand the mechanism of drug release from polymeric nanoparticles and the drug/and nanoparticles loaded hydrogels, the kinetics of drug release was studied by using ten different models namely, zero order, first order, Higuchi, Korsemeyer–Peppas, Hixson–Crowell, Hopfenberg, Baker–Lonsdale, Makoid Banakar, Weibull and Gompertz models. The suitable drug release kinetics model was determined by comparing the best curve fitting and highest correlation coefficient values^71–74^.

#### In vitro drug diffusion study using the Franz model

In vitro diffusion was performed for the hydrogels (loaded with CIP and 5-NPPP nanoparticles) using a Franz diffusion cell for the estimation of passive diffusion of the molecules across membranes. The number of molecules crossing through an artificial biomimicry semipermeable cellulose ester membrane, (Dialysis membrane-110, pore size 2.4 nm, Himedia, Mumbai) was performed using an in vitro setup. The membrane cut into a circular shape was clamped between the donor and receptor compartments of the Franz diffusion cell (effective surface area 6.38 ± 0.31 cm^2^ and 20 mL capacity) and the hydrogel sample was placed on the upper surface of the membrane on the donor side and added with 0.5 mL of phosphate buffer pH 5.8 to mimic the surface pH. The receptor compartment was filled with phosphate buffer pH 7.4 to imitate the systemic pH environment, together maintained at 37 °C temperature. The entire setup was placed on a magnetic stirrer at 100 rpm while withdrawing samples from the receptor side port at regular intervals of time and replacing an equal volume of fresh buffer for a period of 24 h to maintain the perfect sink condition. The diffused drug content was measured using a UV–Visible spectrophotometer at 271.5 nm and 304.5 nm for CIP and 5-NPPP, respectively. The experiments were performed in triplicates, and the average values with the standard deviations are reported^71^.

#### Acute dermal toxicity

The prepared drug-loaded hydrogels were immersed in 0.6 M of NaCl solution for 30 min and were wiped off with tissue paper for complete dryness before placing it on the animal skin. The in vivo studies were performed after obtaining approval from the Institutional Animal Ethical Committee of SASTRA Deemed to be University (Approval number: 696/SASTRA/IAEC/RPP). The guidelines for the usage of animals for the experiments were followed strictly under supervision. The study involved the usage of male and female Balb/c mice. Evaluation of dermal toxicity was carried out as per the protocol of OECD guidelines^61^. Female and male mice in the age group of 8–16 weeks, weighing between 14–21 g and 18–24 g, respectively were taken for the study. Approximately 24 h before the study, the weight of the animals was noted, and hair was removed from the dorsal area by trimming. The test hydrogels were placed on the dorsal region ensuring good contact with the skin and bandaged to retain the gel in contact. The animal was observed twice every day for a total period of 14 days (n = 5/group). The mice were euthanized by CO_2_ inhalation. The group was categorized as given in Table 5.

**Table 5:** Group details for the evaluation of dermal toxicity.

Group number	Treatment	No. of animals (female)
01	Nanoparticles (5-NPPP) loaded hydrogel	05
02	Ciprofloxacin + 5-NPPP nanoparticles loaded hydrogel	05

#### Evaluation of drug efficacy through drug-loaded hydrogels

In this evaluation, both the female and male mice in the age group of 8–16 weeks, weighing between 14–21 g and 18–24 g, respectively, were taken for the study. Animals were divided into groups having equivalent means prior to the infection and treatment. Mice were administered subcutaneously with *S. aureus* 1199B of CFU ~ 10^6^/mL and were left for 24 h. The mice were individually treated with 5-NPPP nanoparticles loaded hydrogel and dual drug-loaded hydrogels (CIP + 5-NPPP nanoparticles) in different treatment groups. The mice were observed regularly for weight and abscess measurement (n = 6/group) using calipers. The abscessed area (mm^2^) was calculated using:

$${\text{Abscess area }} = \, \Pi /2 \, \times \, \left[ {\left( {\text{length of the abscess}} \right) \times \left( {\text{width of the abscess}} \right)} \right]$$

The flat ulcer area (mm^2^) will be calculated using the formulae:

$${\text{Ulcer}}\;{\text{area}} = \, \left( {{\text{length}}\;{\text{of}}\;{\text{ the}}\;{\text{ ulcer}}} \right) \, \times \, \left( {{\text{width }}\;{\text{of}}\;{\text{ the}}\;{\text{ ulcer}}} \right)$$

At the end of the observation on the 7th day, the animals were euthanized by CO_2_ inhalation, and skin samples were taken for histopathological analysis^75–77^. The groups are detailed in Table 6.

**Table 6:** Group details for evaluation of in vivo efficacy of drug-loaded hydrogels.

Group number	Treatment	No. of male mice	No. of female mice
01	Control	03	03
02	Challenge (diseased control)	03	03
03	Challenge + 5-NPPP (low dose)	03	03
04	Challenge + 5-NPPP (medium dose)	03	03
05	Challenge + 5-NPPP (high dose)	03	03
06	Challenge + CIP-loaded hydrogel	03	03
07	Challenge + (5-NPPP + CIP) loaded hydrogel	03	03

#### In vivo serial passage assay

In vivo serial Passage Assay was carried out as described earlier^76,77^. Briefly, early exponential phase culture of *S. aureus* (~ 10^6^ cfu/mL, 50 μL) was first injected into male and female BALB/c mice flanks. Two challenged groups (I and II) were chosen for the study. The challenged group I and II were treated with CIP (0.5 μg/mL) and a combination of 5-NPPP (0.5 μg/mL) and CIP (0.5 μg/mL), respectively (Table 7, Supplementary Fig. 6). The abscess area was excised from the euthanized mice after 24 h. The excised tissue was bead beaten in cell culture grade 1 × PBS and plated onto MSA plates. After the incubation period (16 h), a single colony was picked and suspended in 1 × PBS for a cell density of ~ 10^6^ cfu/mL. As mentioned above, these bacteria were again injected into the flank of the following mouse and were treated with CIP-loaded hydrogel and hydrogel with the dual combination. This was repeated consecutively till the 6th mice of each group. After the second cycle, following euthanization and plating in an agar medium, the bacteria were collected and stored as glycerol stock at − 80 °C. The in vivo passaged bacteria with CIP/CIP and 5-NPPP and the non-passaged bacteria were tested for resistance development.

**Table 7:** Group details for the evaluation of in vivo resistance passage.

Group number	Treatment	No. of animals
01	Ciprofloxacin loaded hydrogel	03 (female)
		03 (male)
02	Cip with nanoparticles (5-NPPP) loaded hydrogel	03 (female)
		03 (male)

### Statistical analysis

The statistical analysis was performed for the data obtained from in vitro release and in vitro membrane diffusion studies through a Student *t*-test with a p-value < 0.05 using Graph pad Prism software version 5.

### Animal ethics

Institutional Animal Ethical Committee of SASTRA Deemed to be University (Approval number: 696/SASTRA/IAEC/RPP). The animal experiments reported are found to be in accordance with ARRIVE guidelines.

## Conclusions

In this study, the combinatorial delivery of an antibiotic with an efflux pump inhibitor molecule through a smart drug delivery system was accomplished. Interestingly, CIP was successfully loaded into the PVA hydrogels without significant physicochemical changes and the in vitro sustained release was achieved for 5 days. The development of polymeric nanoparticles of 5-NPPP was intended to alter the drug release behavior, which was further loaded into the hydrogels to reach the expected sustained drug release profile in accordance to provide a synergistic effect of the dual drugs. The 7% PVA hydrogels loaded with CIP and 5-NPPP polymeric nanoparticles (1:1 ratio of drug:Eudragit RSPO polymer with 200–300 nm size and 90% entrapment efficiency) were optimized to provide the release of a drug by Fick’s diffusion mechanism from the polymeric matrix system. The in vivo studies in mice models proved the hydrogels to be safer with the promising reduction in the bacterial load based on the acute dermal toxicity and efficacy studies. Conclusively, the formulated hydrogels with dual molecules, an antibiotic, and an efflux pump inhibitor, could be recommended clinically for treating soft skin infections with minimal resistance and improved patient compliance.

## Supplementary Information

Supplementary Information.

## Data Availability

The datasets generated during and/or analysed during the current study are available from the corresponding author on reasonable request.
