# Supplementary material for: Polymer based dual drug delivery system for targeted treatment of fluoroquinolone resistant Staphylococcus aureus mediated infections
Source: Sci Rep. 2023 Jul 14;13:11373. doi: 10.1038/s41598-023-38473-3 (PMC10349073; doi:10.1038/s41598-023-38473-3)
Supplement: Supplementary file 1 — Supplementary Information. [file 41598_2023_38473_MOESM1_ESM.docx]

**Polymer based dual drug delivery system for targeted treatment of fluoroquinolone resistant *Staphylococcus aureus* mediated infections**

**Supplementary Data**


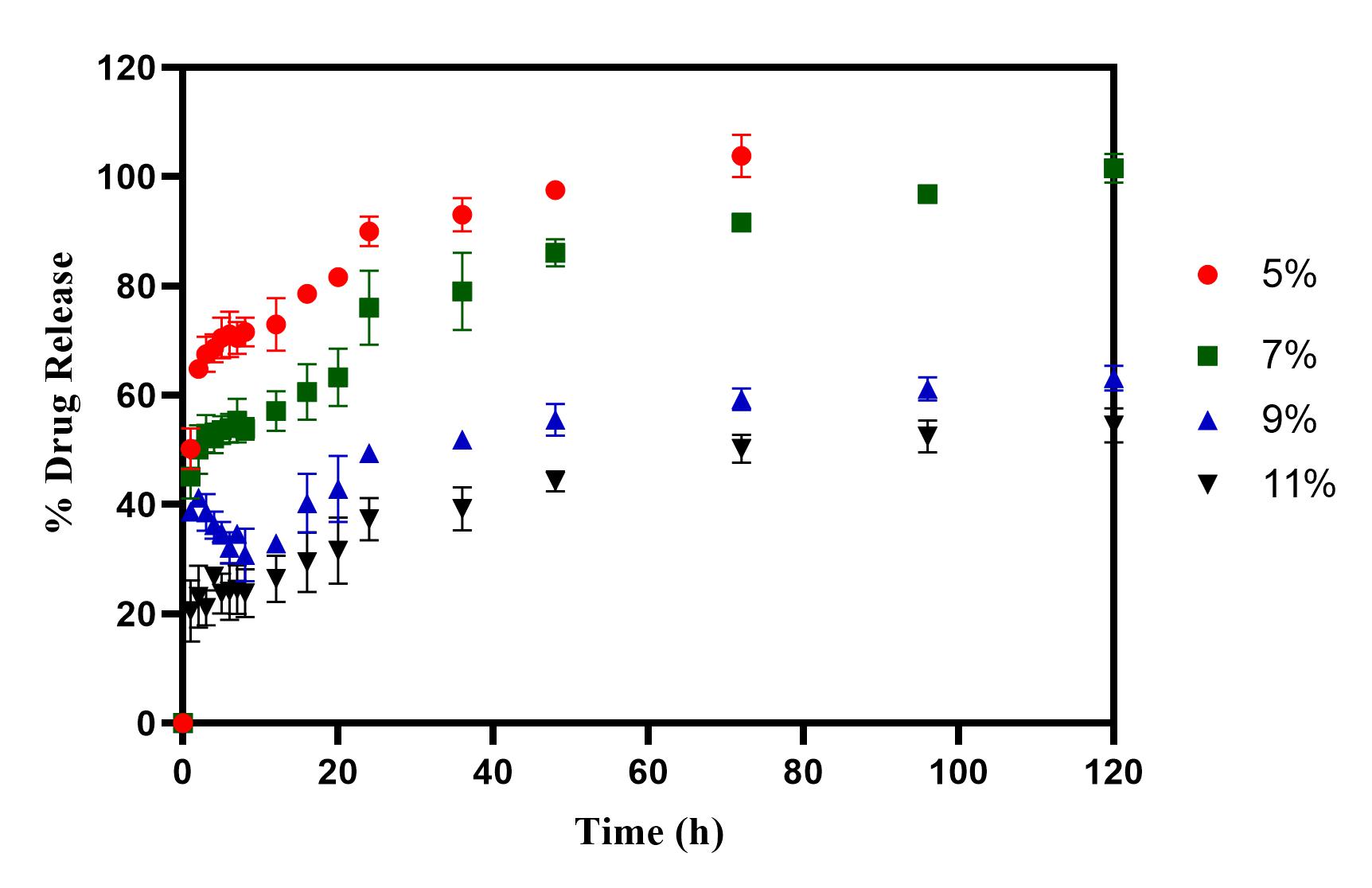
**Supplementary Figure 1: Cumulative Drug Release profile of ciprofloxacin hydrochloride from ciprofloxacin hydrochloride loaded hydrogels at pH 5.8**


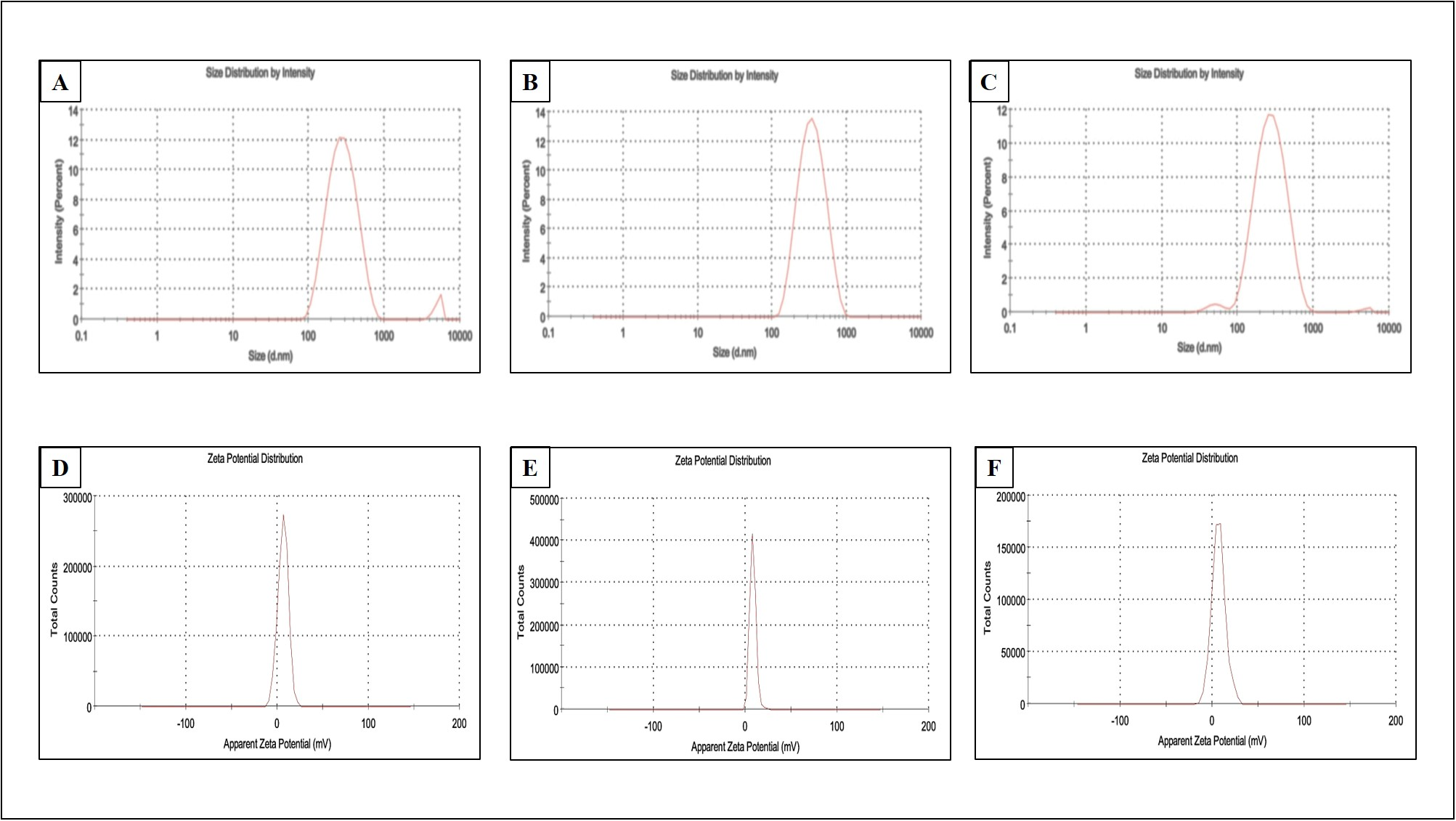


**Supplementary Figure 2: Particle Size Distribution (A) 1:0.5 (B) 1:1 (C) 1:2 and Zeta Potential of (D) 1:0.5 (E) 1:1 (D) 1:2 ratio 5-NPPP Polymeric Nanoparticles**


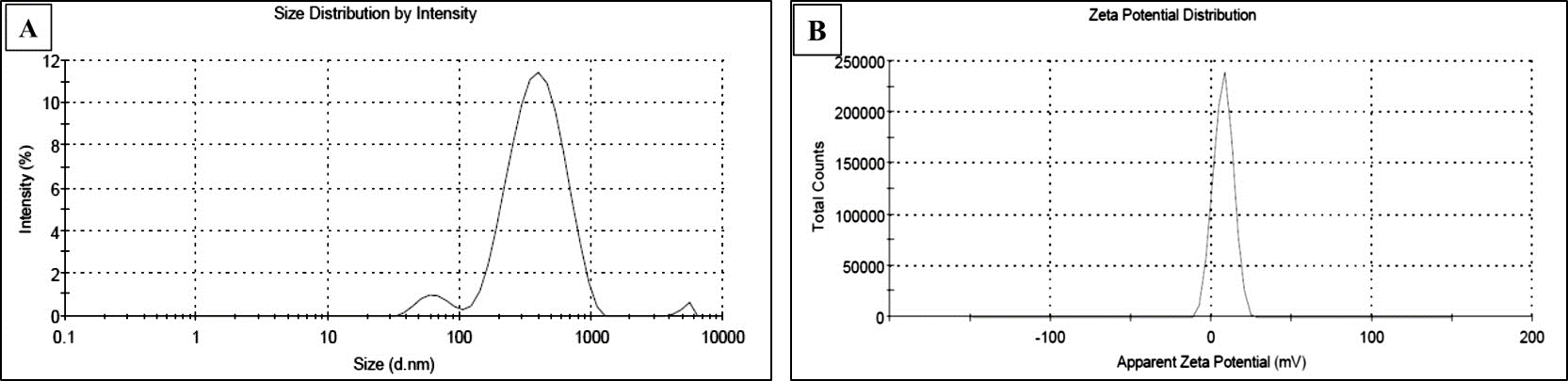


**Supplementary Figure 3: Particle Size Distribution (A) and Zeta Potential of 1:1 5-NPPP Polymeric Nanoparticles after 6 months storage at real-time conditions of 30°C/65%RH**

**
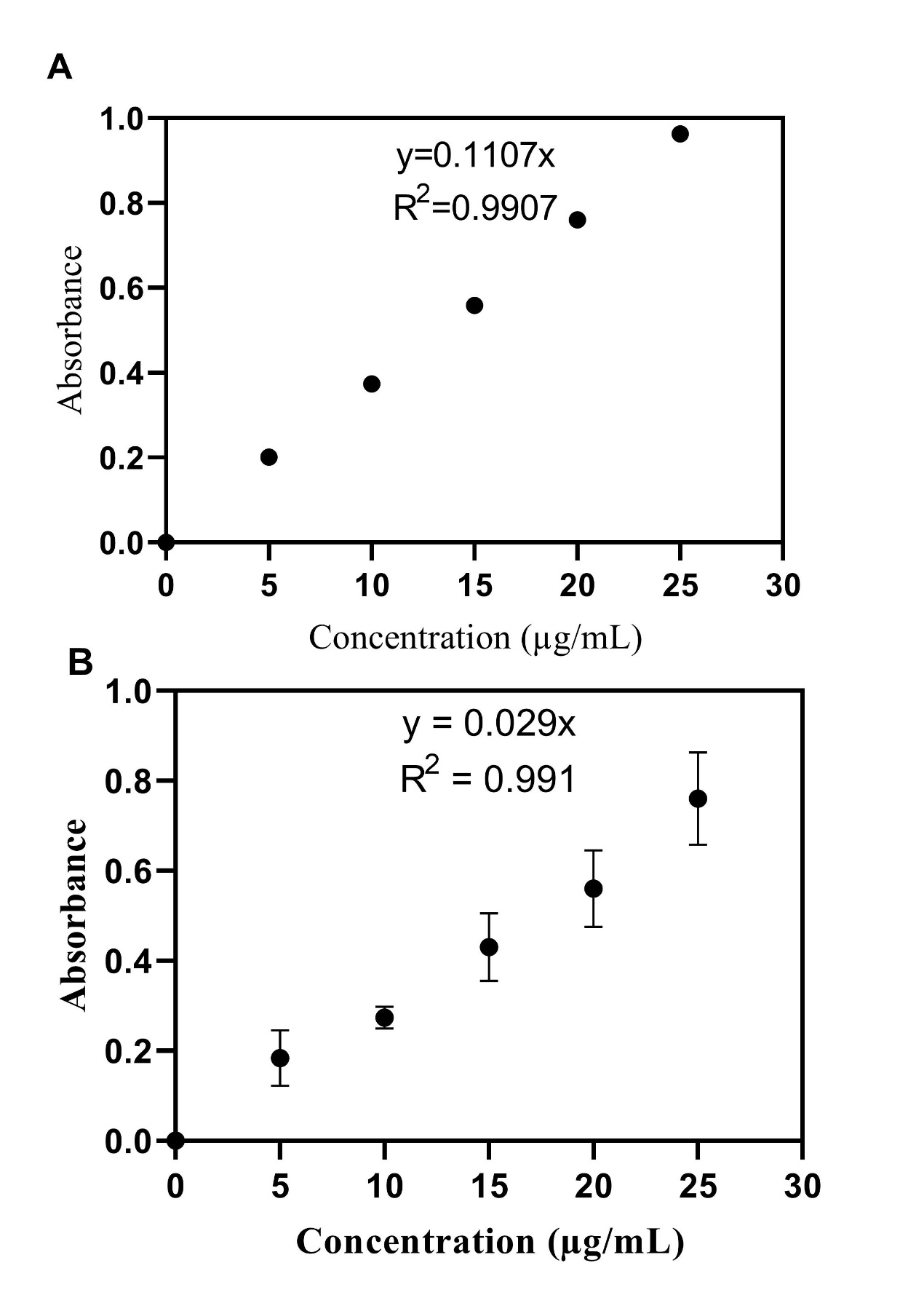
**

**Supplementary Figure 4: Standard Calibration of A) Ciprofloxacin Hcl
B) 5-NPPP in phosphate buffer pH 7.4 using UV-Visible Spectroscopy**


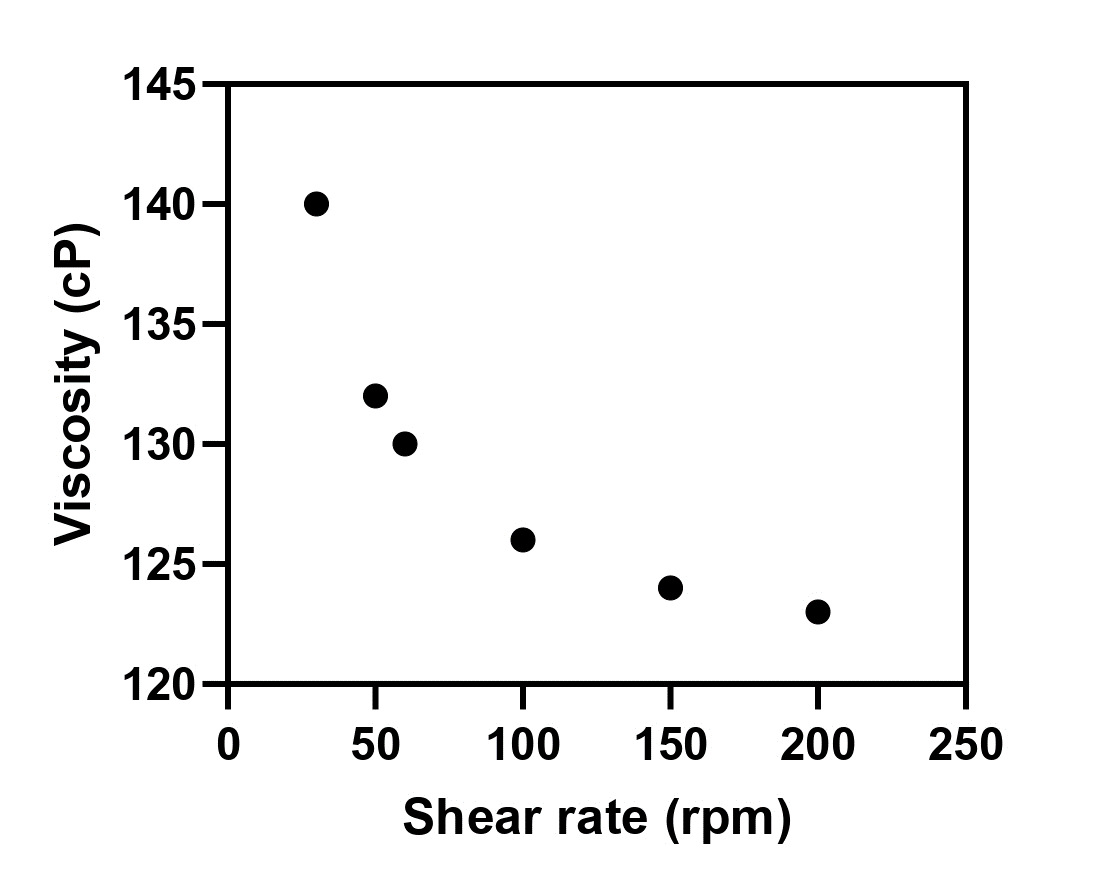


**Supplementary Figure 5: Rheology of the hydrogel forming 7% PVA polymer (n=3, SD<2)**

**
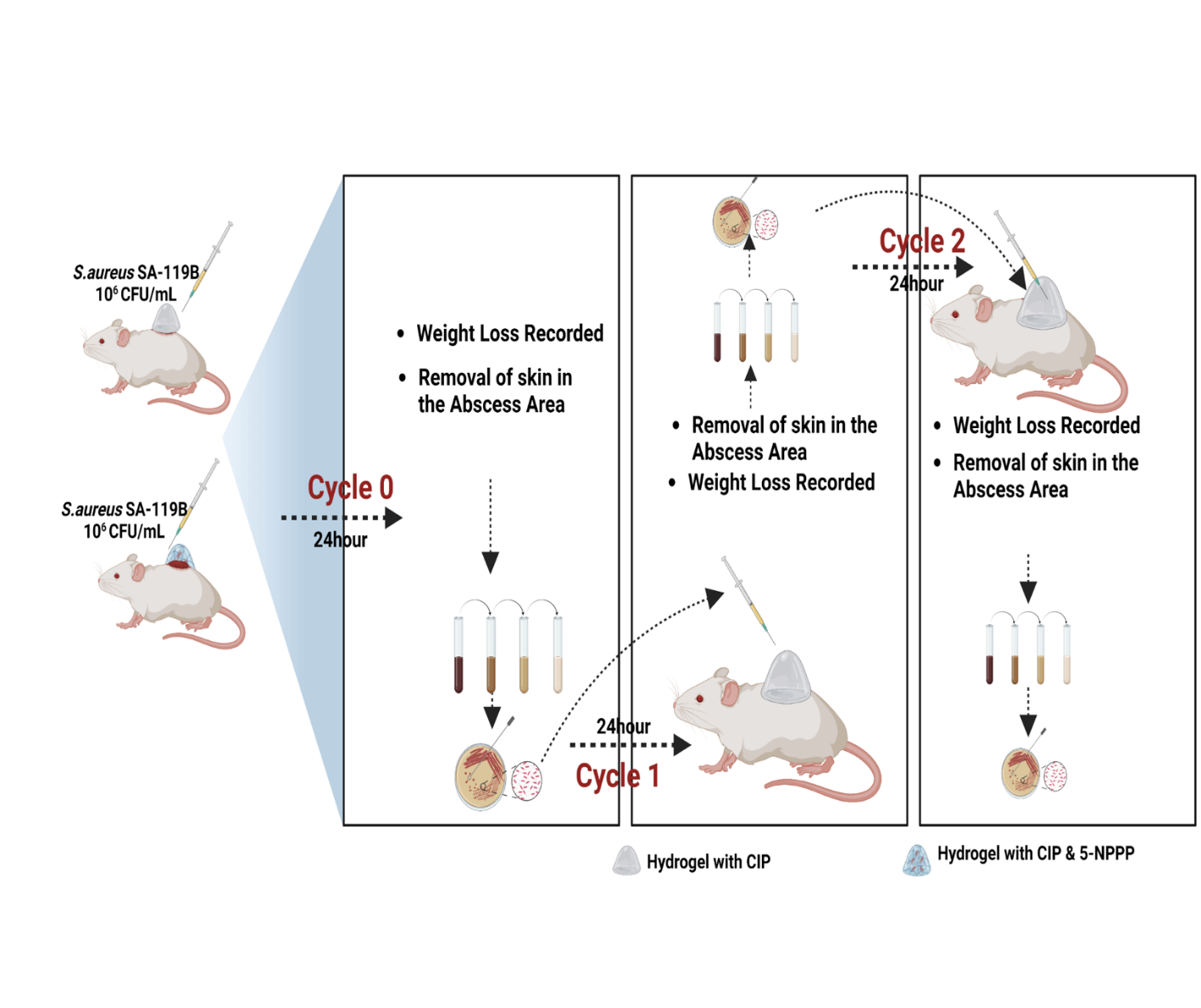
**

**Supplementary Figure 6: Graphical Description of *in-vivo* resistance passage performed on Balb/c mice on both genders of mice.**

**Supplementary Table 1: *In-vitro* drug release kinetics of hydrogels and nanoparticles**

| **Kinetics Model** | **Parameters** | **Ciprofloxacin 7% Hydrogel** | **5-NPPP Nanoparticles 7% hydrogels** | **1:1 ratio 5-NPPP nanoparticles** |
| --- | --- | --- | --- | --- |
| **Zero order** | **K_0_** | 1.157 | 1.113 | 2.915 |
|  | **R^2^** | 0.3712 | 0.4839 | 0.4074 |
|  | **SS** | 16497.6983 | 15788.8500 | 7656.1141 |
| **First order** | **K_1_** | 0.061 | 0.057 | 0.089 |
|  | **R^2^** | 0.8070 | 0.7328 | 0.9826 |
|  | **SS** | 2313.3364 | 2843.4176 | 221.7613 |
| **Higuchi** | **K_H_** | 11.102 | 10.718 | 16.257 |
|  | **R^2^** | 0.7732 | 0.7405 | 0.9577 |
|  | **SS** | 2718.6407 | 2760.7587 | 537.8492 |
| **Korsemeyer-Peppas** | **K_KP_** | 23.872 | 23.926 | 19.441 |
|  | **R^2^** | 0.9941 | 0.9960 | 0.9684 |
|  | **n** | 0.303 | 0.293 | 0.441 |
|  | **SS** | 70.6484 | 42.4220 | 402.6827 |
| **Hixson-Crowell** | **K_HC_** | 0.013 | 0.012 | 0.025 |
|  | **R^2^** | 0.6545 | 0.5754 | 0.9504 |
|  | **SS** | 4140.9272 | 4517.5689 | 630.5531 |
| **Hopfenberg** | **K_HB_** | 0.0001 | 0.0001 | 0.0001 |
|  | **R^2^** | 0.8070 | 0.7327 | 0.9826 |
|  | **SS** | 2313.8863 | 2844.1062 | 221.8595 |
| **Baker-Lonsdale** | **K_BL_** | 0.004 | 0.004 | 0.007 |
|  | **R^2^** | 0.9682 | 0.9487 | 0.9702 |
|  | **SS** | 380.7404 | 546.2778 | 379.1157 |
| **Makoid-Banakar** | **K_MB_** | 22.143 | 23.211 | 14.106 |
|  | **R^2^** | 0.9967 | 0.9965 | 0.9911 |
|  | **SS** | 39.6956 | 37.5513 | 113.6269 |
| **Weibull** | **R^2^** | 0.9890 | 0.9868 | 0.9943 |
|  | **SS** | 131.9703 | 139.9933 | 72.1845 |
| **Gompertz** | **R^2^** | 0.9487 | 0.9436 | 0.9745 |
|  | **SS** | 615.4648 | 599.6645 | 324.5155 |

**Supplementary Table 2: *In-vivo* behavioral patterns, dermal signs, feed intake and mortality rate of mice from acute dermal toxicity studies – 5-NPPP loaded hydrogels**

| Animal Number | Gender | Abnormal Signs (Skin and fur, eyes, mucous membrane, behavioral patterns, internal organs after necropsy) | Feed Intake for 14 days | Weekly body weight (gm)  n=5 | | | Mortality rate* (%) |
| --- | --- | --- | --- | --- | --- | --- | --- |
|  |  |  |  | Day 0 | Day 7 | Day 14 |  |
| 1 | Female | No change | Good | 29.79 | 30.14 | 30.98 | 0 |
| 2 | Female | No change | Good | 30.14 | 32.31 | 33.33 | 0 |
| 3 | Female | No change | Good | 25.02 | 25.36 | 26.1 | 0 |
| 4 | Female | No change | Good | 24.5 | 26.09 | 27.08 | 0 |
| 5 | Female | No change | Good | 27 | 27.54 | 28 | 0 |

*Mortality rate is the number of dead mice divided by total number of mice

**Supplementary Table 3: *In-vivo* behavioral patterns, dermal signs, feed intake and mortality rate of mice from acute dermal toxicity studies – 5-NPPP nanoparticle and CIP loaded hydrogels**

| Animal Number | Gender | Abnormal Signs (Skin and fur, eyes, mucous membrane, behavioral patterns, internal organs after necropsy) | Feed Intake for 14 days | Weekly body weight (gm)  n=5 | | | Mortality rate* (%) |
| --- | --- | --- | --- | --- | --- | --- | --- |
|  |  |  |  | Day 0 | Day 7 | Day 14 |  |
| 1 | Female | No change | Good | 25.61 | 26.61 | 28.42 | 0 |
| 2 | Female | No change | Good | 25.82 | 27.42 | 28.56 | 0 |
| 3 | Female | No change | Good | 27.24 | 28.36 | 30.12 | 0 |
| 4 | Female | No change | Good | 29.3 | 31.26 | 32.42 | 0 |
| 5 | Female | No change | Good | 28.5 | 29.2 | 29.5 | 0 |

*Mortality rate is the number of dead mice divided by total number of mice
